# Akanthopyrones A–D, α-Pyrones Bearing a 4-*O*-Methyl-β-d-glucopyranose Moiety from the Spider-Associated Ascomycete *Akanthomyces novoguineensis*

**DOI:** 10.3390/molecules22071202

**Published:** 2017-07-18

**Authors:** Wilawan Kuephadungphan, Soleiman E. Helaly, Charuwan Daengrot, Souwalak Phongpaichit, Janet Jennifer Luangsa-ard, Vatcharin Rukachaisirikul, Marc Stadler

**Affiliations:** 1 Department of Microbial Drugs, Helmholtz Centre for Infection Research, 38124 Braunschweig, Germany; wilawan.kue@hotmail.co.th (W.K.); soleiman.helaly@aswu.edu.eg (S.E.H.); 2 Department of Microbiology, Faculty of Science, Prince of Songkla University, Songkhla 90112, Thailand; souwalak.p@psu.ac.th; 3 Department of Chemistry, Faculty of Science, Aswan University, Aswan 81528, Egypt; 4 Department of Chemistry, Faculty of Science, Prince of Songkla University, Songkhla 90112, Thailand; noocharu@gmail.com (C.D.); vatcharin.r@psu.ac.th (V.R.); 5 Natural Products Research Center of Excellence, Prince of Songkla University, Songkhla 90112, Thailand; 6 National Centre for Genetic Engineering and Biotechnology (BIOTEC), Pathumthani 12120, Thailand; jajen@biotec.or.th; 7 Center of Excellence for Innovation in Chemistry, Prince of Songkla University, Songkhla 90112, Thailand

**Keywords:** cordycipitaceae, hypocreales, invertebrate-associated fungi, α-pyrones, 4-*O*-methyl-β-d-glucopyranose

## Abstract

Hypocrealean fungi have proved to be prolific bioactive metabolite producers; they have caught the attention of mycologists throughout the world. However, only a few studies on the insect and spider parasitic genus *Akanthomyces* have so far been carried out. In this study, we report the isolation, structural elucidation and biological activities of four unprecedented glycosylated α-pyrone derivatives, akanthopyrones A–D (**1**–**4**), from a culture of *Akanthomyces novoguineensis* collected in Thailand. The chemical structures of the akanthopyrones were determined by extensive 1D- and 2D-NMR, and HRMS spectroscopic analysis. Their absolute configurations were determined. Akanthopyrone A (**1**) exhibited weak antimicrobial activity against *Bacillus subtilis* DSM10 and cytotoxicity against the HeLa cell line KB-3-1, while akanthopyrone D (**4**) showed weak activity against *Candida tenuis* MUCL 29892.

## 1. Introduction

Fungi are well-known as prolific producers of biologically active secondary metabolites [1]. In particular, the pathogenic fungi associated with invertebrates have been proved to be an untapped source of structurally diverse novel substances with various biological activities [2,3]. These fungi can degrade the insect’s cuticle containing high densities of chitin by enzyme secretion and utilize the host as their nutrient source [4]. Invertebrate pathogenic fungi can also produce various kinds of secondary metabolites in order to overcome the host’s defense as well as to defeat other competing microorganisms [2]. One of these interesting invertebrate-associated fungal genera is *Akanthomyces*, which was established for *A. aculeatus* Lebert, infecting moths found in Europe [5] and is presently classified in the Cordycipitaceae. To date, more than 10 species of *Akanthomyces* associated with insects or spiders have been described and accepted worldwide [5,6,7,8,9]. Remarkably, only a few studies on *Akanthomyces* have been carried out so far in order to investigate and explore its novel secondary metabolites that may be useful in agricultural and medicinal applications [10,11,12,13].

**Figure 1:**
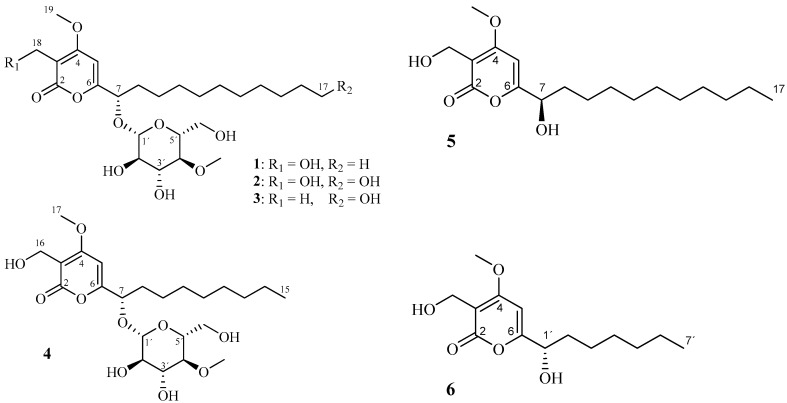
Structures of akanthopyrones A–D (**1**–**4**) and similar pyrones reported in literature (**5**–**6**).

In the course of a study on invertebrate-pathogenic fungi collected from the tropical rainforest in Thailand, a collection of spider-associated fungal specimens was encountered in order to generate their HPLC profiles. Compounds possessing a 4-methylglucose moiety have rarely been found in natural sources, except for some insect pathogenic fungi [14,15,16,17,18,19,20]. In the current study, we report the isolation, structure elucidation, absolute configuration and biological activity of four new α-pyrone derivatives bearing a 4-*O*-methyl-β-d-glucopyranose, for which we propose the trivial names, akanthopyrones A–D (**1**–**4**; Figure 1), from the spider-associated fungus *A. novoguineensis*. The current study is the second publication on the secondary metabolites of this species. In a concurrent paper, the distribution pattern of secondary metabolites in the genus has been compared, five other new metabolites of the fungus were reported, and akanthopyrones were produced as main compounds and proved to be species-specific secondary metabolites [21].

## 2. Results and Discussion

**Figure 2:**
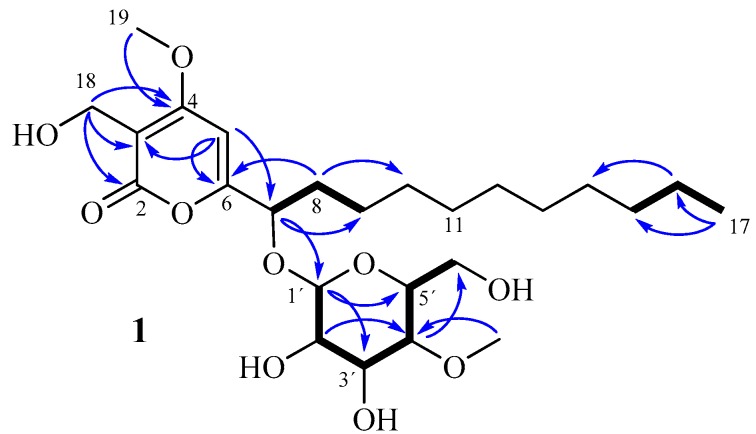
Selected COSY (bold bonds) and HMBC (blue arrows) correlations for akanthopyrone A (**1**).

The main compound of this family, Compound **1**, was obtained as a pale brown gum. HRMS of **1** afforded the molecular ion cluster at *m*/*z* 503.2851 [M + H]^+^ indicating the molecular formula of C_25_H_42_O_10_ (calcd. 503.2851) with 5 degrees of unsaturation. The planar structure of **1** was assigned by the analysis of its NMR data (Table 1) as follows. A series of COSY correlations from the oxygenated methine 7-H to 8-H_2_, 8-H_2_ to 9-H_2_ and between the methylenes 15-H_2_, 16-H_2_, and 17-H_3_ together with resonances of 10 protons to 5 methylene carbons in the HSQC spectrum of **1** allowed the construction of undecane alkyl chain (C-7/C-17). Furthermore, HMBC correlations from the olefinic proton 5-H to C-3/C-6, from the oxygenated methylene H_2_-18 to C-2/C-3/C-4 and from the methoxy group H_3_-19 to C-4 established a 3-hydroxymethyl-4-methoxy-2*H*-pyranone substructure. The undecane chain was connected to C-6 of the α-pyrone ring by HMBC correlations from 7-H to C-6/C-5 and from 5-H to C-7 (Figure 2). The remaining seven C-atoms were assigned to a methylated glucose unit. Starting from a characteristic anomeric proton 1’-H at δ_H_ 4.29 and δ_C_ 100.6, a series of ^1^H-^1^H-COSY and ^1^H-^13^C-HMBC correlations assigned the sugar unit as 4-*O*-methyl-glucopyranose. The position of the methoxy group (δ_H_ 3.57) on the sugar moiety was evident from HMBC correlation to C-4′. Finally, the mutual ^1^H and ^13^C correlation through the glycoside bond between the anomeric methine and the oxymethine group C-7 allowed the determination of the attachment of the glycosyl residue at 7-O (Figure 2). Consequently, compound **1** was determined as the first member of a group of α-pyrone derivatives bearing 4-*O*-methyl-β-d-glucopyranose, for which we propose the trivial name akanthopyrone A (**1**).

The relative configuration of akanthopyrone A (**1**) was provided by NOESY data and coupling constant analysis. Starting from a typical 1′-H signal of a β-glycoside at δ_H_ 4.29 with *J*_1′,2′_ = 7.6 Hz (a signal at δ_H_ ~ 5.2 with coupling constant of 3.6 Hz is expected in the case of α-glycoside) [22]. Its β-glycoside connection was evident by a ^1^*J*_C,H_ coupling of 160 Hz for the anomeric methine. A chain of vicinal *trans* couplings of *J*_2′,3′_ = 9.2 Hz, *J*_3′,4′_ = 8.8 Hz and *J*_4′,5′_ = 9.5 Hz indicated that the methine protons H-1′ to H-5′ all occupy axial positions. The chair conformation completely agrees with the restrictions set by the NOEs between 1′-H and 3′-H/5′-H. The d-configuration of the sugar was established by comparing the specific rotation of the aqueous layer of its acid hydrolysate (${\left[\alpha \right]}_{\text{D}}^{25}$+45, *c* 0.05, MeOH) with that of 4-*O*-methyl-d-glucopyranose (${\left[\alpha \right]}_{\text{D}}^{25}$+80, *c* 1.3, MeOH) [23]. The organic layer of the acid hydrolysis of **1** contains the aglycone which is identical to our previously reported dalsymbiopyrone (**5**) [24]. Nevertheless, the opposite specific rotations (−33° for **1** and +150° for **5**) indicate the configuration of C-7 in **1** to be (*S*). In addition, the specific rotation of **1** was similar to that for dothiodeopyrone (**6**) [25], a related pyrone with *S*-configuration at C-7 (${\left[\alpha \right]}_{\text{D}}^{25}$−77, *c* 0.22, CHCl_3_).

**Figure 3:**
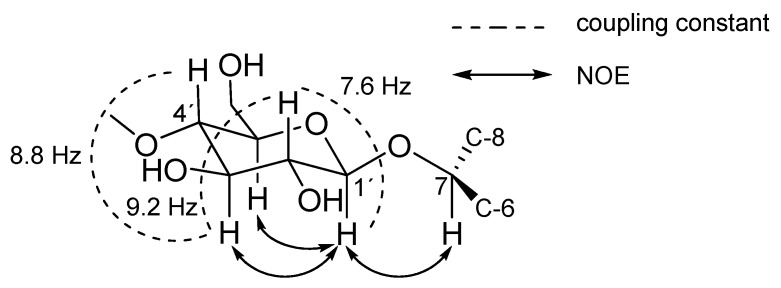
Partial view showing the absolute configuration of **1**.

**Figure 4:**
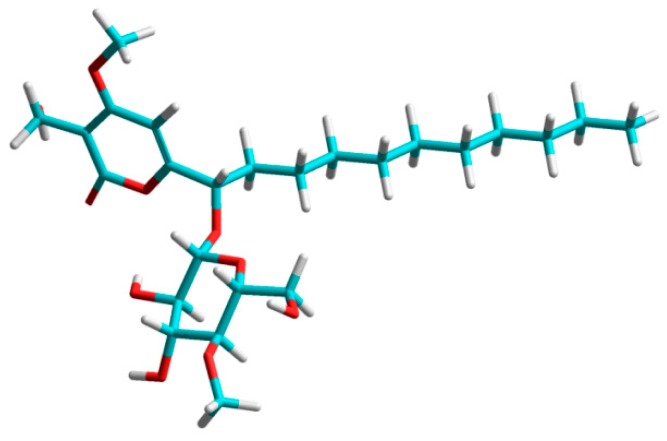
Energy minimized conformation of akanthopyrone A (**1**) using HyperChem, pm3 calculation method.

The absolute configuration was further confirmed by applying the rule reported by Seo et al. [26]. Compared to dalsymbiopyrone (**5**), the signal of C-7 was shifted by 5.2 ppm downfield, the typical glycosidation shift of an α-carbon. Additionally, the glycosidation induces distinct β-shifts in the aglycone. Since the β-shifts mainly reflect the chirality of the aglycone, this provides a valuable tool for assignment of the absolute configuration of the glycosylated carbons [26]. According to Seo et al. [26]**,** the absolute value of ∆δ_C_ = δ_glycoside_ − δ_aglycone_ for the β-carbon *anti* to the pyranose-ring oxygen is always larger than that for the β-carbon *syn* to the oxygen. In the case of akanthopyrone A (**1**), a shift of −2.5 ppm for C-6 and −1.5 ppm for C-8 together with a strong NOE between H-7 and H-1′ indicated that C-6 is *anti* to the pyranose oxygen (Figure 3). Thus, the absolute configuration of akanthopyrone A (**1**) must be 7*S*, 1′*S*, 2′*R*, 3′*R*, 4′*S*, 5′*R*, as shown in a model calculated for akanthopyrone (**1**) using HyperChem (Ver. 8.0.10); pm3 calculation method showing the minimized energy 3D structure of **1** is shown in Figure 4.

Akanthopyrone B (**2**) was obtained as a brown gum. Its molecular formula was determined by HRMS as C_25_H_42_O_11_. Compared to **1**, the molecular formula of **2** includes an additional oxygen atom. ^1^H- and ^13^C-NMR data of akanthopyrone A (**1**) were largely preserved in **2**, the new derivative was assigned as the alcohol derivative of akanthopyrone A (**1**) based on the comparison of its NMR data with those of **1**. COSY and HMBC correlations furnished the additional hydroxy group at C-17 of the undecane chain of **2**. NOESY data and coupling constant values for the sugar moiety in **2** were identical to those of **1** (Table 1), indicating the same relative configuration for **2**. The co-occurrence suggests that **2** possess the same absolute configuration as **1** (7*S*, 1′*S*, 2′*R*, 3′*R*, 4′*S*, 5′*R*).

Akanthopyrone C (**3**) was isolated as a brown gum as well. HRMS data revealed the molecular formula of C_25_H_42_O_10_ (calcd 503.2851) calculated from the ion peak at *m*/*z* 503.2852 [M + H]^+^. Compound **3** shared the same molecular formula with **1**; nevertheless, its ^1^H- and ^13^C-NMR data (Table 1) were more consistent with those of **2**. Comprehensive analysis of the NMR data revealed that **3** comprise the same hydroxyl undecane chain as **2**. Nevertheless, the oxymethylene signals in **2** (C-18) were replaced by signals for the singlet methyl group at δ_H_ 1.87 and δ_C_ 8.6. HMBC correlations from 18-H_3_ to C-2/C-3/C-4 indicated the attachment of a methyl group to the *sp*^2^ quaternary carbon C-3 of the pyrone ring instead of the oxygenated methylene in the case of **1** and **2**. Therefore, akanthopyrone C (**3**) was determined as a new member of the akanthopyrones family, which shared the relative configuration with **1** and **2** based on NOESY data, coupling constant values. The co-occurrence suggests that **3** possesses the same absolute configuration as **1** (7*S*, 1′*S*, 2′*R*, 3′*R*, 4′*S*, 5′*R*).

Akanthopyrone D (**4**) was isolated as a brown gum; it possesses the molecular formula C_23_H_38_O_10_ (calcd 457.2432) calculated from the ion peak at *m*/*z* 457.2439 [M + H − H_2_O]^+^. The molecular formula of **4** lacks a C_2_H_4_ fragment compared to **1** which suggested a shorter alkyl chain in **4**. The NMR data of the sugar moiety and the α-pyrone substructure of **1** were largely preserved in **4**. The NMR data of **4** revealed the presence of 9 carbon alkyl chain (nonane chain) assigned from the COSY and HMBC correlations. Thus, akanthopyrone D (**4**)—a new member of the akanthopyrones—shared the relative and the absolute configuration with the other akanthopyrones.

Natural products bearing an α-pyrone moiety are widespread and occur in microbes, fungi plants and invertebrate animals [27]. The pyrone moiety is well-known to play an important role in various types of biological processes as a defense against other organisms and key biosynthetic intermediates as well as metabolites [27]. The α-pyrone is considered as a broad-spectrum and promising bioactive compound—even some of the simplest derivatives exhibit remarkable biological effects [28,29].

In the current study, akanthopyrones A–D (**1**–**4**) were screened for antimicrobial, cytotoxic, anti-biofilm and nematicidal activities. Akanthopyrone A (**1**) exhibited weak antimicrobial activity against *Bacillus subtilis* DSM10 with an MIC value of 300 μg/mL and weak cytotoxicity against HeLa cell line KB-3-1 with an IC50 value of 25 μg/mL while akanthopyrone D (**4**) was active against only *Candida tenuis* MUCL 29892 at an MIC value of 150 μg/mL. However, none of them possessed anti-biofilm or nematicidal activities, even at the highest tested concentration of 33.33 and 100 μg/mL, respectively. These results are consistent with a report of Fairlamb et al. [28] in which the antimicrobial and cytotoxic activities of diverse α-pyrone analogues were evaluated. Most of them showed an inhibitory effect against *B. subtilis* while more than 50% of the tested compounds displayed anti-*C. albicans* effect. Furthermore, they also pointed out that the activity of α-pyrone derivatives in the alkyl class noticeably changed according to the length of the alkyl chain. The longer the alkyl chain, the higher the potency of antimicrobial activity and vice versa on the cytotoxic effect. Weak activity against *B. subtilis* ATCC 6633 at 100 µg/mL was previously reported for the most related pyrone to akanthopyrone A (**1**), dalsymbiopyrone (**5**), and no activity at all concentrations up to that concentration against *Escherichia coli* K12 and *Pichia anomala* was observed for **5**. It inhibited *Colletotrichum gloeosporioides* at 25 µg/mL [24]. Miaolienone, a closely related compound to akanthopyrone A (which however, lacks the sugar moiety), was isolated from the bacterium, *Actinomadura miaoliensis* and has been reported to be an effective TNF-α inhibitor [30].

Recently, *A. novoguineensis* was also found to be a producer of a well-known immunosuppressant drug, mycophenolic acid [10]. This indicates that this species could be a promising source of further novel biologically active secondary metabolites.

## 3. Materials and Methods

### 3.1. General

1D and 2D NMR spectra were recorded on a Bruker (Bremen, Germany) Avance III 700 spectrometer with a 5 mm TXI cryoprobe (^1^H 700 MHz, ^13^C 175 MHz) and a Bruker Avance III 500 (^1^H 500 MHz, ^13^C 125 MHz) spectrometer; optical rotations were measured on a Perkin-Elmer 241 polarimeter. All HPLC-MS analyses were performed on Agilent 1260 Infinity Systems (Santa Clara, CA, USA) with a diode array detector and C_18_ Acquity UPLC BEH column (2.1 × 50 mm, 1.7 μm) from Waters with the gradient described by Noumeur et al. [31] combined with ion trap MS (amazon speed, Bruker, Bremen, Germany), and HR-ESIMS spectra on a time-of-flight (TOF) MS (Maxis, Bruker, Germany). Chemicals and solvents were obtained from AppliChem GmbH (Darmstadt, Germany), Avantor Performance Materials (Deventor, Netherlands), Carl Roth GmbH & Co. KG (Karlsruhe, Germany), and Merck KGaA (Darmstadt, Germany) in analytical and HPLC grade.

### 3.2. Fungal Material

A spider-associated fungal specimen of *A. novoguineensis* was collected from Ton-Nga-Chang Wildlife Sanctuary, Thailand and the culture was deposited at the Department of Microbiology, Faculty of Science, Prince of Songkla University and BIOTEC Culture Collection (BCC), Pathum Thani, Thailand as BCC47894. Its 5.8S/ITS nrDNA was sequenced following the protocol described by Luangsa-ard et al. [32] and submitted to GenBank (accession number JX192691). The species description is provided in the Supplementary Material.

### 3.3. Fermentation and Extraction

Twenty mycelial plugs (0.5 × 0.5 cm^2^) were cut from actively growing colonies maintained on potato dextrose agar (PDA) and inoculated into a 30 × 500 mL Erlenmeyer flask containing 150 mL of potato dextrose broth (PDB) supplemented with 0.1% of yeast extract. After incubation at room temperature (RT) under static condition for ten weeks, the culture filtrate was recovered by vacuum filtration and subsequently extracted according to the procedure described by Phainuphong et al. [33].

### 3.4. Isolation of Compounds ***1**–**4***

The fermented broth of the fungus BCC47894 was extracted with 4 L of ethyl acetate to give the oily residue which was subsequently dissolved with methanol and fractionated using an Agilent 1100 series HPLC system (Agilent Technologies, Wilmington, DE, USA). The reverse-phase C18 column (Kromasil 250 × 20 mm, 7 μm; MZ Analysentechnik, Mainz, Germany) was used as the stationary phase and the mobile phase was composed of deionized water (Milli-Q, Millipore, Schwalbach, Germany, solvent A) and acetonitrile (ACN, HPLC-grade, solvent B). The separation was carried out according to following gradient: linear from 20% to 80% solvent B in 30 min; afterwards, linear gradient to 100% solvent B in 2 min; thereafter, isocratic conditions at 100% for 5 min, with a flow rate of 20 mL/min. UV detection was carried out at 210, 280 and 354 nm and fractions were collected and combined according to the observed peaks. Compound **1** (18.3 mg) was obtained at a retention time *t*_R_ = 20–21 min, Compound **2** (4.5 mg) at *t*_R_ = 10–11 min, Compound **3** (2 mg) at *t*_R_ = 13–14 min and Compound **4** (2 mg) at *t*_R_ = 15–16 min.

***Akanthopyrone A*** **(1):** brown gum; ${\left[\alpha \right]}_{\text{D}}^{25}$−52° (*c* 1.7, MeOH); ^1^H-NMR and ^13^C-NMR see Table 1; LCMS *m*/*z* 485.20 [M + H − H_2_O]^+^ (73), 309 [M + H − (4-methylglucose)]^+^, 969 [2M + Na]^+^ (9), 547 [M − H + HCOOH]^−^ (75), 1003 [2M − H]^−^ (100); HRESIMS *m*/*z* 503.2851 [M + H]^+^ (calcd. for C_25_H_43_O_10_^+^, 503.2851).

***Akanthopyrone B* (2):** brown gum; ${\left[\alpha \right]}_{\text{D}}^{25}$−54° (*c* 0.3, MeOH); ^1^H-NMR and ^13^C NMR see Table 1; LCMS *m*/*z* 501 [M + H − H_2_O]^+^ (100), 325 [M + H − (4-methylglucose)]^+^, 1001 [2M + H − 2H_2_O]^+^ (35), 517 [M − H]^−^ (10), 563 [M − H + HCOOH]^−^ (100); HRESIMS *m*/*z* 519.2801 [M + H]^+^ (calcd. for C_25_H_43_O_11_^+^, 519.2800).

***Akanthopyrone C* (3):** brown gum; ${\left[\alpha \right]}_{\text{D}}^{25}$−17° (*c* 0.1, MeOH); ^1^H-NMR and ^13^C-NMR see Table 1; LCMS *m/z* 503 [M + H]^+^ (100), 525 [M + Na]^+^ (13), 327 [M + H − (4-methylglucose)]^+^, 501 [M − H]^−^ (12), 547 [M − H + HCOOH]^−^ (100); HRESIMS *m*/*z* 503.2852 [M + H]^+^ (calcd. for C_25_H_43_O_10_^+^, 519.2851).

***Akanthopyrone D* (4):** brown gum; ${\left[\alpha \right]}_{\text{D}}^{25}$−24° (*c* 0.16, MeOH); ^1^H-NMR and ^13^C-NMR see Table 1; LCMS *m*/*z* 457 [M + H − H_2_O]^+^ (100), 281 [M + H − (4-methylglucose)]^+^, 497 [M + Na]^+^ (12), 473 [M − H]^−^ (4), 519 [M − H + HCOOH]^−^ (100); HRESIMS *m*/*z* 457.2434 [M + H − H_2_O]^+^ (calcd. for C_23_H_37_O_9_^+^, 457.2432).

### 3.5. Hydrolysis of Akanthopyrone A *(**1**)*

Compound **1** (1.5 mg) was hydrolyzed with 10% aqueous HCl (1 mL) at 90 °C for 12 h. The reaction mixture was then diluted with H_2_O (2 mL) and extracted with EtOAc (2 × 3 mL). The aqueous layer was concentrated under vacuum to yield 4-*O*-methyl-d-glycopyranose (${\left[\alpha \right]}_{\text{D}}^{25}$+45, *c* 0.05, MeOH). The organic layer was evaporated to dryness under reduced pressure to obtain the aglycone (0.8 mg) (${\left[\alpha \right]}_{\text{D}}^{25}$−33, *c* 0.08, MeOH).

### 3.6. Biological Activities

#### 3.6.1. Antimicrobial Activity Assay

The minimum inhibitory concentration (MIC) of Compound **1**–**4** was determined using the broth microdilution method according to Richter et al. [34] against *B. subtilis* DSM10, *E. coli* DSM498, *C. tenuis* MUCL29892 and *Mucor plumbeus* MUCL49355. Stock suspension of each bacterium and yeast (100 μL) was transferred to 100 mL of EBS medium and yeast-malt-glucose (YMG) medium, respectively. Suspensions of *B. subtilis* and *C. tenuis* were incubated on a rotary shaker at 30 °C for 18–24 h while *E. coli* was grown at 37 °C for 24 h. After incubation, the suspension was adjusted to a concentration of 6.7 × 10^5^ cells/mL using a hemacytometer. The spore suspension of *M. plumbeus* was prepared at a concentration of 6.7 × 10^5^ conidia/mL using YMG medium. The determination of MIC was performed in a 96-well microtiter plate. The compounds dissolved in methanol at a concentration of 4.5 mg/mL (20 μL) were transferred to the first row of the plate. Standard antibiotics including ciprofloxacin (bacteria) and nystatin (yeast and fungus), and methanol were used as positive and negative controls, respectively. Inoculum suspension (280 μL) was added to the first row containing compounds and 150 μL was added to the rest. The solutions were then serially 2-fold diluted to 8 concentrations ranging from 2.34 to 300 μg/mL. Plates were incubated at 30 °C on a microplate-vibrating shaker for 24 h for bacteria and 48 h for yeast and filamentous fungus. After incubation, the lowest concentration of each compound at which no visible growth was observed and recorded as the MIC.

#### 3.6.2. Cytotoxicity Activity Assay

The isolated compounds were tested for cytotoxicity using the MTT (3-(4,5-dimethylthiayol-2-yl)-2,5-diphenyltetrayolium bromide) method in 96-well microtiter plates following the procedure previously described by Richter et al. [34] against the cervix carcinoma cell line KB-3-1 (a HeLa derivative) and the established mouse fibroblast cell line L929.

#### 3.6.3. Anti-biofilm Activity Assay

The ability of akanthopyrones to inhibit biofilm formation of *Staphylococcus aureus* DSM1104 (ATCC25923) and *Pseudomonas aeruginosa* PA14 [35] was evaluated using the microtiter dish biofilm formation assay described by Helaly et al. [21].

#### 3.6.4. Nematicidal Activity Assay

The determination of nematicidal activity of new compounds against *Caenorhabditis elegans* was performed in a 24-microwell plate using a microwell plate assay slightly modified from the method reported by Stadler et al. [36]. The free-living nematode, *C. elegans*, was monoxenically cultured on nematodes agar (soy peptone 2 g, NaCl 1 g, Agar 20 g, 1000 mL of distilled water; after autoclaving, the following ingredients were added: cholesterol (1 mg/mL EtOH) 0.5 mL, 1M CaCl_2_ 1 mL, 1M MgSO_4_ 1 mL, 40 mM potassium phosphate buffer 12.5 mL, pH 6.8) with living *E. coli* DSM498, at 20 °C in the dark for a week. After incubation, adult nematodes were suspended in sterile distilled water and the nematode suspension was then adjusted to give a concentration of 500 nematodes/mL. Four concentrations of 100, 50, 20 and 10 μg/mL of each compound were tested (total volume 1 mL/well). Standard nematicide, ivermectin and 1% MeOH were used as the positive inhibitory control and solvent control, respectively. The plate was incubated at 20 °C in the dark and nematicidal activity was recorded after 18 h of incubation.

## 4. Conclusions

Akanthopyrones A–D (**1**–**4**)—four new α-pyrone derivatives, bearing a 4-*O*-methyl-β-d-glucopyranose—were obtained from a spider-associated fungus *A. novoguineensis*. The major compound akanthopyrone A (**1**) exhibited weak antimicrobial activity against *B. subtilis* DSM10 and cytotoxicity against HeLa cell line KB-3-1, while akanthopyrone D (**4**) showed weak activity against *C. tenuis* MUCL 29892. This finding demonstrates that *Akanthomyces* constitutes a rich, hitherto untapped source for novel metabolites.

## Figures and Tables

**Table 1:** ^1^H- and ^13^C-NMR data for akanthopyrones A–D (**1**–**4**, Methanol-*d*_4_).

Pos	1 ^a^		2		3		4	
	δ_H_ (*J* in Hz)	δ_C_, Type	δ_H_ (*J* in Hz)	δ_C_, Type	δ_H_ (*J* in Hz)	δ_C_, Type	δ_H_ (*J* in Hz)	δ_C_, Type
2	-	165.1, C	-	167.1, C	-	167.9, C	-	167.8, C
3	-	104.4, C	-	105.1, C	-	102.1, C	-	105.1, C
4	-	167.4, C	-	170.8, C	-	169.0, C	-	170.8, C
5	6.76, s	94.9, CH	7.06, s	96.6, CH	7.00, s	96.8, CH	7.06, s	96.6, CH
6	-	165.3, C	-	167.8, C	-	164.9, C	-	167.0, C
7	4.51, ^b^ dd (7.3, 4.7)	76.4, CH	4.67, dd (7.3, 4.6)	77.1, CH	4.65, dd (7.5, 4.9)	77.0, CH	4.66, dd (7.3, 4.7)	77.1, CH
8	1.74, m; 1.84, m	34.1, CH_2_	1.76, m; 1.86, m	35.4, CH_2_	1.76, m; 1.85, m	35.4, CH_2_	1.76, m; 1.86, m	35.4, CH_2_
9	1.38, m	24.9, CH_2_	1.44, m	25.9, CH_2_	1.43, m	25.9, CH_2_	1.43, m	25.9, CH_2_
10	1.29, ^b^ m	29.2, CH_2_	1.35, ^b^ m	30.6, CH_2_	1.35, ^b^ m	30.5, CH_2_	1.35, ^b^ m	30.5, CH_2_
11	1.27, ^b^ m	29.3,^c^ CH_2_	1.32, ^b^ m	30.7, ^c^ CH_2_	1.32, ^b^ m	30.6,^c^ CH_2_	1.32, ^b^ m	30.6, ^c^ CH_2_
12	1.25, ^b^ m	29.4,^c^ CH_2_	1.30, ^b^ m	30.9, ^c^ CH_2_	1.30, ^b^ m	30.7,^c^ CH_2_	1.30, ^b^ m	30.7, ^c^ CH_2_
13	1.25, ^b^ m	29.5,^c^ CH_2_	1.30, ^b^ m	30.8, ^c^ CH_2_	1.30, ^b^ m	30.9,^c^ CH_2_	1.30, ^b^ m	33.2, CH_2_
14	1.25, ^b^ m	29.6,^c^ CH_2_	1.30, ^b^ m	30.7, ^c^ CH_2_	1.30, ^b^ m	30.8,^c^ CH_2_	1.30, ^b^ m	23.9, CH_2_
15	1.25, ^b^ m	31.8, CH_2_	1.34, ^b^ m	27.1, CH_2_	1.35, ^b^ m	27.1, CH_2_	0.90, t (7.1)	14.6, CH_3_
16	1.29, ^b^ m	22.6, CH_2_	1.52, m	33.8, CH_2_	1.53, m	33.8, CH_2_	4.44, s	54.2, CH_2_
17	0.87, t (6.9)	14.1, CH_3_	3.54, t (6.7)	63.2, CH_2_	3.54, t (6.8)	63.2, CH_2_	3.99, s	58.0, CH_3_
18	4.51, ^b^ s	54.3, CH_2_	4.44, s	54.2, CH_2_	1.87, s	8.6, CH_3_	-	-
19	3.93, s	56.9, CH_3_	3.99, s	58.1, CH_3_	3.96, s	57.7, CH_3_	-	-
**β-4-*O*-methyl-d-glucopyranose**								
1′	4.29, d (7.6)	100.6, CH	4.26, d (7.9)	102.7, CH	4.25, d (7.9)	102.6, CH	4.25, d (7.9)	102.7, CH
2′	3.43, dd (7.6, 9.2)	73.6, CH	3.29, dd (7.9, 9.2)	75.3, CH	3.28, dd (7.9, 9.2)	75.4, CH	3.28, dd (7.9, 9.2)	75.4, CH
3′	3.54, dd (9.2, 8.8)	76.6, CH	3.43, dd (9.2, 8.8)	78.3, CH	3.43, dd (9.1, 9.1)	78.3, CH	3.43, dd (9.1, 9.1)	78.4, CH
4′	3.21, dd (8.8, 9.5)	79.1, CH	3.09, dd (9.2, 9.5)	81.1, CH	3.09, dd (9.1, 9.5)	81.1, CH	3.09, dd (9.1, 9.5)	81.1, CH
5′	3.26, m	75.4, CH	3.23, m	77.4, CH	3.22, m	77.4, CH	3.22, m	77.4, CH
6′	3.74, dd (12.2, 4.3) 3.89, dd (12.2, 2.4)	61.8, CH_2_	3.68, dd (11.8, 5.3) 3.84, dd (11.8, 2.0)	62.5, CH_2_	3.68, dd (11.8, 5.4) 3.83, dd (11.8, 2.0)	62.5, CH_2_	3.68, dd (11.8, 5.4) 3.83, dd (11.8, 2.0)	62.5, CH_2_
OMe	3.57, s	60.8, CH_3_	3.56, s	60.9, CH_3_	3.55, s	60.9, CH_3_	3.55, s	61.0, CH_3_

**1** and **2** were measured at 500 MHz (^13^C 125 MHz); **3** and **4** were measured at 700 MHz spectrometer (^13^C 175 MHz); ^a^ in CDCl_3_, ^b^ overlapping signals, ^c^ assignment may be interchanged.

## Supplementary Materials

The species description of the producers, UV, HRESIMS and ^1^H, ^13^C, COSY, HSQC, HMBC NMR spectra of Compounds **1**–**4** are available as Supplementary Material.

## References

[1] Karwehl S., Stadler M. (2016). Exploitation of Fungal Biodiversity for Discovery of Novel Antibiotics. Curr. Top. Microbiol. Immunol..

[2] Molnár I., Gibson D.M., Krasnoff S.B. (2010). Secondary metabolites from entomopathogenic Hypocrealean fungi. Nat. Prod. Rep..

[3] Isaka M., Kittakoop P., Kirtikara K., Hywel-Jones N.L., Thebtaranonth Y. (2005). Bioactive substances from insect pathogenic fungi. Acc. Chem. Res..

[4] Humber R.A. (2008). Evolution of entomopathogenicity in fungi. J. Invertebr. Pathol..

[5] Mains E.B. (1950). Entomogenous Species of *Akanthomyces*, *Hymenostilbe* and *Insecticola* in North America. Mycol. Soc. Am..

[6] Hsieh L.S., Tzean S.S., Wu W.J. (1997). The genus *Akanthomyces* on spiders from Taiwan. Mycol. Soc. Am..

[7] Hywel-Jones N. (1996). *Akanthomyces* on spiders in Thailand. Mycol. Res..

[8] Samson R.A., Brady B.L. (1982). *Akanthomyces novoguineensis* sp. nov.. Trans. Br. Mycol. Soc..

[9] Samson R.A., Evans H.C. (1974). Notes on entomogenous fungi from Ghana. II. The genus *Akanthomyces*. Plant Biol..

[10] Kinoshita H., Wongsuntornpoj S., Ihara F., Nihira T. (2016). Anti-*Rhodotorula* activity of mycophenolic acid enhanced in the presence of polyene-antibiotic nystatin. Lett. Appl. Microbiol..

[11] Kuephadungphan W., Phongpaichit S., Luangsa-ard J.J., Rukachaisirikul V. (2013). Antimicrobial activity of invertebrate-pathogenic fungi in the genera *Akanthomyces* and *Gibellula*. Mycoscience.

[12] Lee S.Y., Nakajima I., Ihara F., Kinoshita H., Nihira T. (2005). Cultivation of entomopathogenic fungi for the search of antibacterial compounds. Mycopathologia.

[13] Wagenaar M.M., Gibson D.M., Clardy J. (2002). Akanthomycin, a new antibiotic pyridone from the entomopathogenic fungus *Akanthomyces gracilis*. Org. Lett..

[14] Asai T., Yamamoto T., Oshima Y. (2012). Aromatic polyketide production in *Cordyceps indigotica*, an entomopathogenic fungus, induced by exposure to a histone deacetylase inhibitor. Org. Lett..

[15] Kikuchi H., Takahashi N., Oshima Y. (2004). Novel aromatics bearing 4-O-methylglucose unit isolated from the oriental crude drug *Bombyx Batryticatus*. Tetrahedron Lett..

[16] Isaka M., Palasarn S., Supothina S., Komwijit S., Luangsa-Ard J.J. (2011). Bioactive compounds from the scale insect pathogenic fungus *Conoideocrella tenuis* BCC 18627. J. Nat. Prod..

[17] Kornsakulkarn J., Thongpanchang C., Lapanun S., Srichomthong K. (2009). Isocoumarin glucosides from the scale insect fungus *Torrubiella tenuis* BCC 12732. J. Nat. Prod..

[18] Kornsakulkarn J., Saepua S., Srichomthong K., Supothina S., Thongpanchang C. (2012). New mycotoxins from the scale insect fungus *Aschersonia coffeae* Henn. BCC 28712. Tetrahedron.

[19] Saepua S., Kornsakulkarn J., Choowong W., Supothina S., Thongpanchang C. (2015). Bioxanthracenes and monomeric analogues from insect pathogenic fungus *Conoideocrella luteorostrata* Zimm. BCC 31648. Tetrahedron.

[20] Seephonkai P., Isaka M., Kittakoop P., Boonudomlap U., Thebtaranonth Y. (2004). A novel ascochlorin glycoside from the insect pathogenic fungus *Verticillium hemipterigenum* BCC 2370. J. Antibiot..

[21] Helaly S.E., Kuephadungphan W., Phongpaichit S., Luangsa-ard J.J., Rukachaisirikul V., Stadler M. (2017). Five unprecedented secondary metabolites from the spider parasitic fungus *Akanthomyces novoguineensis*. Molecules.

[22] Giner J.L., Feng J., Kiemle D.J. (2016). NMR tube degradation method for sugar analysis of glycosides. J. Nat. Prod..

[23] Smith F. (1951). The constitution of mesquite gum. Part III. The structure of the monomethyl glucuronic acid component. J. Chem. Soc..

[24] Pažoutová S., Follert S., Bitzer J., Keck M., Surup F., Šrůtka P., Holuša J., Stadler M. (2013). A new endophytic insect-associated *Daldinia* species, recognised from a comparison of secondary metabolite profiles and molecular phylogeny. Fungal Divers..

[25] Chomcheon P., Wiyakrutta S., Sriubolmas N., Ngamrojanavanich N., Mahidol C., Ruchirawat S., Kittakoop P. (2009). Metabolites from the endophytic mitosporic Dothideomycete sp. LRUB20. Phytochemistry.

[26] Seo S., Tomita Y., Tori K., Yoshimura Y. (1978). Determination of the absolute configuration of a secondary hydroxy group in a chiral secondary alcohol using glycosidation shifts in carbon-13 nuclear magnetic resonance spectroscopy. J. Am. Chem. Soc..

[27] McGlacken G.P., Fairlamb I.J.S. (2005). 2-Pyrone natural products and mimetics: Isolation, characterisation and biological activity. Nat. Prod. Rep..

[28] Fairlamb I.J.S., Marrison L.R., Dickinson J.M., Lu F.J., Schmidt J.P. (2004). 2-Pyrones possessing antimicrobial and cytotoxic activities. Bioorg. Med. Chem..

[29] Helaly S., Schneider K., Nachtigall J., Vikineswary S., Tan G.Y.A., Zinecker H., Imhoff J.F., Süssmuth R.D., Fiedler H.-P. (2009). Gombapyrones, new α-pyrone metabolites produced by *Streptomyces griseoruber* Acta 3662. J. Antibiot..

[30] Tseng M., Su Y.-S., Cheng M.-J., Liu T.-W., Chen I.-S., Wu M.-D., Chang H.-S., Yuan G.-F. (2013). Chemical constituents from a soil-derived Actinomycete, *Actinomadura miaoliensis* BCRC 16873, and their inhibitory activities on lipopolysaccharide-induced tumor necrosis factor production. Chem. Biodivers..

[31] Noumeur S.R., Helaly S.E., Jansen R., Gereke M., Stradal T.E.B., Harzallah D., Stadler M. (2017). Preussilides A–F, bicyclic polyketides from the endophytic fungus *Preussia similis* with antiproliferative activity. J. Nat. Prod..

[32] Luangsa-ard J.J., Mongkolsamrit S., Thanakitpipattana D., Khonsanit A., Tasanathai K., Noisripoom W., Humber R.A. (2017). Clavicipitaceous entomopathogens: New species in *Metarhizium* and a new genus *Nigelia*. Mycol. Prog..

[33] Phainuphong P., Rukachaisirikul V., Saithong S., Phongpaichit S., Bowornwiriyapan K., Muanprasat C., Srimaroeng C., Duangjai A., Sakayaroj J. (2016). Lovastatin analogues from the soil-derived fungus *Aspergillus sclerotiorum* PSU-RSPG178. J. Nat. Prod..

[34] Richter C., Helaly S.E., Thongbai B., Hyde K.D., Stadler M. (2016). Pyristriatins A and B: Pyridino-cyathane antibiotics from the Basidiomycete *Cyathus* cf. *striatus*. J. Nat. Prod..

[35] Rahme L.G., Stevens E.J., Wolfort S.F., Shao J., Tompkins R.G., Ausubel F.M. (1995). Common virulence factors for bacterial pathogenicity in plants and animals. Science.

[36] Stadler M., Anke H., Arendholz W.-R., Hansske F., Anders U., Sterner O., Bergquist K.-E. (1993). Lachnumon and lachnumol A, new metabolites with nematicidal and antimicrobial activities from the ascomycete *Lachnum papyraceum* (Karst.) Karst. J. Antibiot..

