# Supplementary material for: Akanthopyrones A–D, α-Pyrones Bearing a 4-O-Methyl-β-d-glucopyranose Moiety from the Spider-Associated Ascomycete Akanthomyces novoguineensis
Source: Molecules. 2017 Jul 18;22(7):1202. doi: 10.3390/molecules22071202 (PMC6152309; doi:10.3390/molecules22071202)
Supplement: Supplementary file 1 [file molecules-22-01202-s001.pdf]

Supplementary Materials

Akanthopyrones A – D, α-pyrones bearing a 4-*O*-methyl-*β*-D-glucopyranose moiety from the spider-associated ascomycete *Akanthomyces novoguineensis*

Wilawan Kuephadungphan[1,2,a], Soleiman E. Helaly[1,3,a], Charuwan Daengrot[4], Souwalak Phongpaichit[2,5], Janet Jennifer Luangsa-ard[6], Vatcharin Rukachaisirikul[4,7], Marc Stadler[1,*]

Affiliation

[1] Department of Microbial Drugs, Helmholtz Centre for Infection Research, Braunschweig 38124, Germany

[2]Department of Microbiology, Faculty of Science, Prince of Songkla University, Songkhla 90112, Thailand

[3]Department of Chemistry, Faculty of Science, Aswan University, Aswan 81528, Egypt

[4]Department of Chemistry, Faculty of Science, Prince of Songkla University, Songkhla 90112, Thailand

[5]Natural Products Research Center of Excellence, Prince of Songkla University, Songkhla 90112, Thailand

[6]National Centre for Genetic Engineering and Biotechnology (BIOTEC), Pathumthani 12120, Thailand

[7] Center of Excellence for Innovation in Chemistry, Prince of Songkla University, Songkhla 90112, Thailand

Correspondence

Prof. Dr. Marc Stadler
Department of Microbial Drugs, Helmholtz Centre for Infection Research, Inhoffenstrasse 7, 38124 Braunschweig, Germany
E-mail: marc.stadler@helmholtz-hzi.de
Phone: +49 531 6181-4240
Fax: +49 531 6181-9499

[a] These authors contributed equally to this work

Isolation and characterization of fungal material

The fungal specimen was found on underside of living leaf of the forest plant which was carefully picked away and kept in plastic box. The collected specimenwas immediately isolated into pure culture by gentle swipe small agar plug of potato dextrose agar (PDA) containing 50 mg/L penicillin and streptomycin over the spores located on synnemata using a fine sterile needle. Agar plugs with spores were placed on PDA containing antibiotics which was then incubated at 25ºC and the conidial germination was observed daily as well as fungal contamination. Pure culture was isolated onto fresh PDA plate without antibiotics by hyphal tip isolation, allowed to grow for 4-6 weeks and subsequently deposited to Prince of Songkla University and BIOTEC, Thailand with the BCC code no. BCC47894.

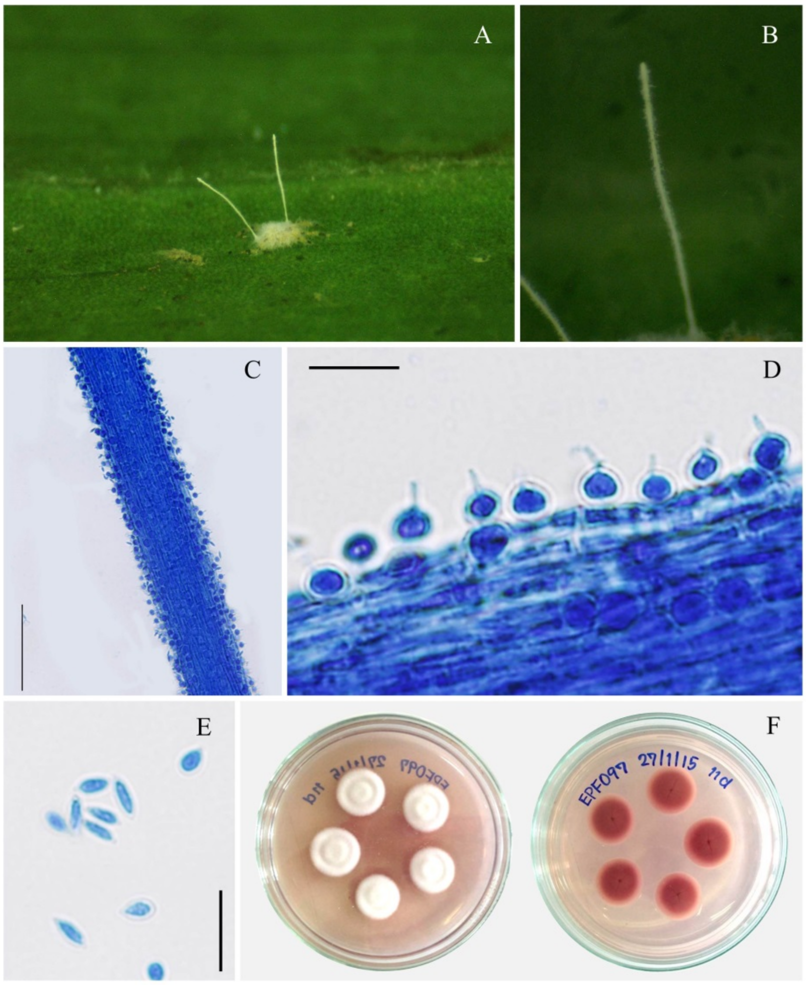

Figure S1. Morphological characteristics of *A. novoguineensis* strain BCC47894(A) and its colonies on PDA at 25 °C for 11 days (F), part of synnema showing phialides (B-D), conidia (E), Scale bar: C = 500 μm, D-E = 10 μm

The akanthopyrone producer was characterized as *Akanthomyces novoguineensis* according to Samson and Brady[1], Hywel-Jones[2] and Hsieh et al. [3]by producing a couple of creamish white, erect, cylindrical synnemata on the host (Figure 1). The globose, hyaline, smooth-walled phialides, eachwith a single distinct

neck were located scatteringly along the entire length of synnema (Figure S1). The conidia forming at the tip of the neck were hyaline and ellipsoid (Figure S1). On PDA,the white colonies were slow-growing, reaching a diameter of 0.8-1.0 cm in 11 days at 25˚C. The colony reverse was pink with diffused pale pink pigment in the agar (Figure S1). Sporulation was not found.

To support the morphological species identification, the ITS rDNA sequence of the producer was performed nucleotide-nucleotide search using the Basic Local Alignment Search Tool (BLAST) from the National Center for Biotechnology Information (NCBI, USA). The BLAST search with the query sequence hit sequences mostly belonged to *A. novoguineensis* and *A. novoguineensis* strain BCC41865 (GenBank accession no. JN201872) was the top BLAST match with 97% identity.

Based on morphological features and the BLAST search result, the species could be identified as *A. novoguineensis*.

Table S1 Biological activities of Compound **1-4**

**Test strains**	**1**	**2**	**3**	**4**	**Reference**
Antimicrobial activity		MIC (µg/ml)			µg/ml
Bacillus subtilis DSM10	300	n.a.	n.a.	n.a.	0.78[a]
Escherichia coli DSM498	n.a.	n.a.	n.a.	n.a.	0.78[a]
Candida tenuis MUCL29892	n.a.	n.a.	n.a.	150	0.78[b]
Mucor plumbeus MUCL49355	n.a.	n.a.	n.a.	n.a.	6.25[b]
Cytotoxicity		IC50 (µg/ml)			µM
Cervix carcinoma cell line KB-3-1	25	n.a.	n.a.	n.a.	0.00022[c]
Mouse fibroblast cell line L929	n.a.	n.a.	n.a.	n.a.	0.0038[d]
Nematicidal activity (µg/ml)					
Caenorhabditis elegans	n.a.	n.a.	n.a.	n.a.	7.5[e]
Anti-biofilm activity (µg/ml)					
Staphylococcus aureus DSM1104	n.a.	n.a.	n.a.	n.a.	1000[f]
Pseudomonas aeruginosa PA14	n.a.	n.a.	n.a.	n.a.	1000[f]

n.a. no activity, [a] ciprofloxacin, [b] nystatin, [c] epothilone B, [d] epothilone A, [e] ivermectin [f] tetracycline

References

1. Samson, R. A.; Brady, B. L. Akanthomyces novoguineensis sp. nov. *Trans. Br. Mycol. Soc.***1982**, *79,* 571–572.

2. Hywel-Jones, N. Akanthomyces on spiders in Thailand. *Mycol. Res.***1996**, *100*, 1065–1070.

3. Hsieh, L. S.; Tzean, S. S.; Wu, W. J. The genus Akanthomyces on spiders from Taiwan. *Mycol. Soc. Am.***1997**, *89*, 319–324.

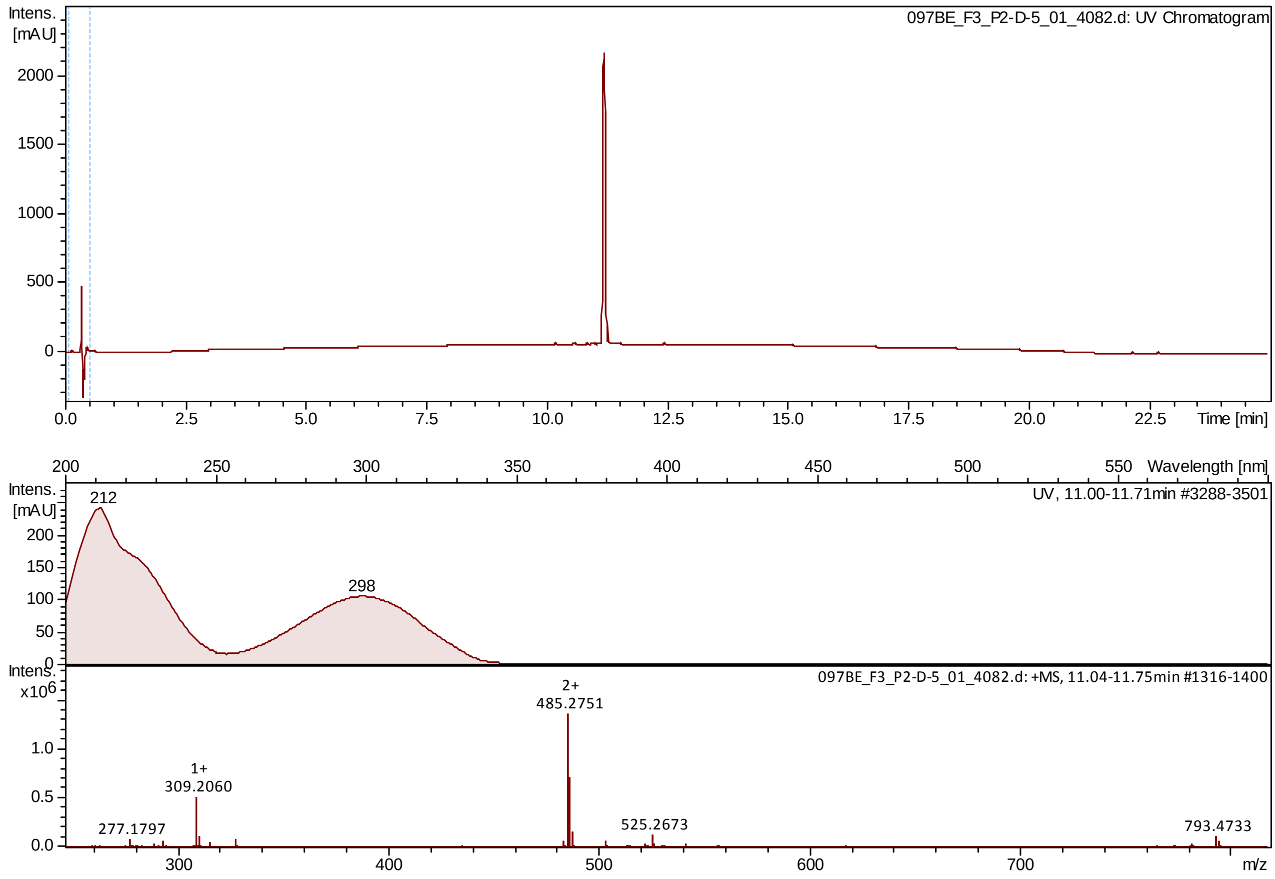

Figure S2. HR-MS chromatograms for akanthopyrone A (**1**)

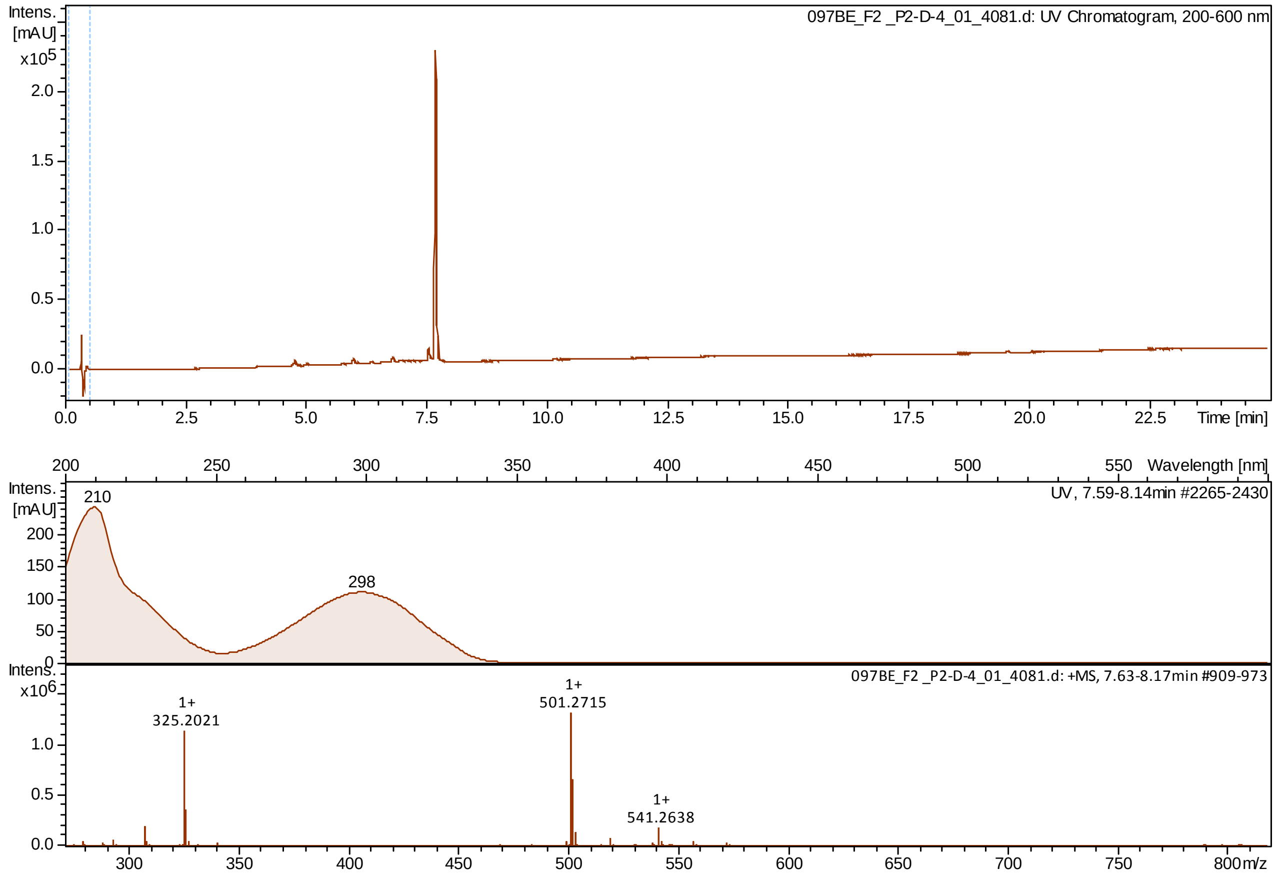

Figure S3. HR-MS chromatograms for akanthopyrone B (**2**)

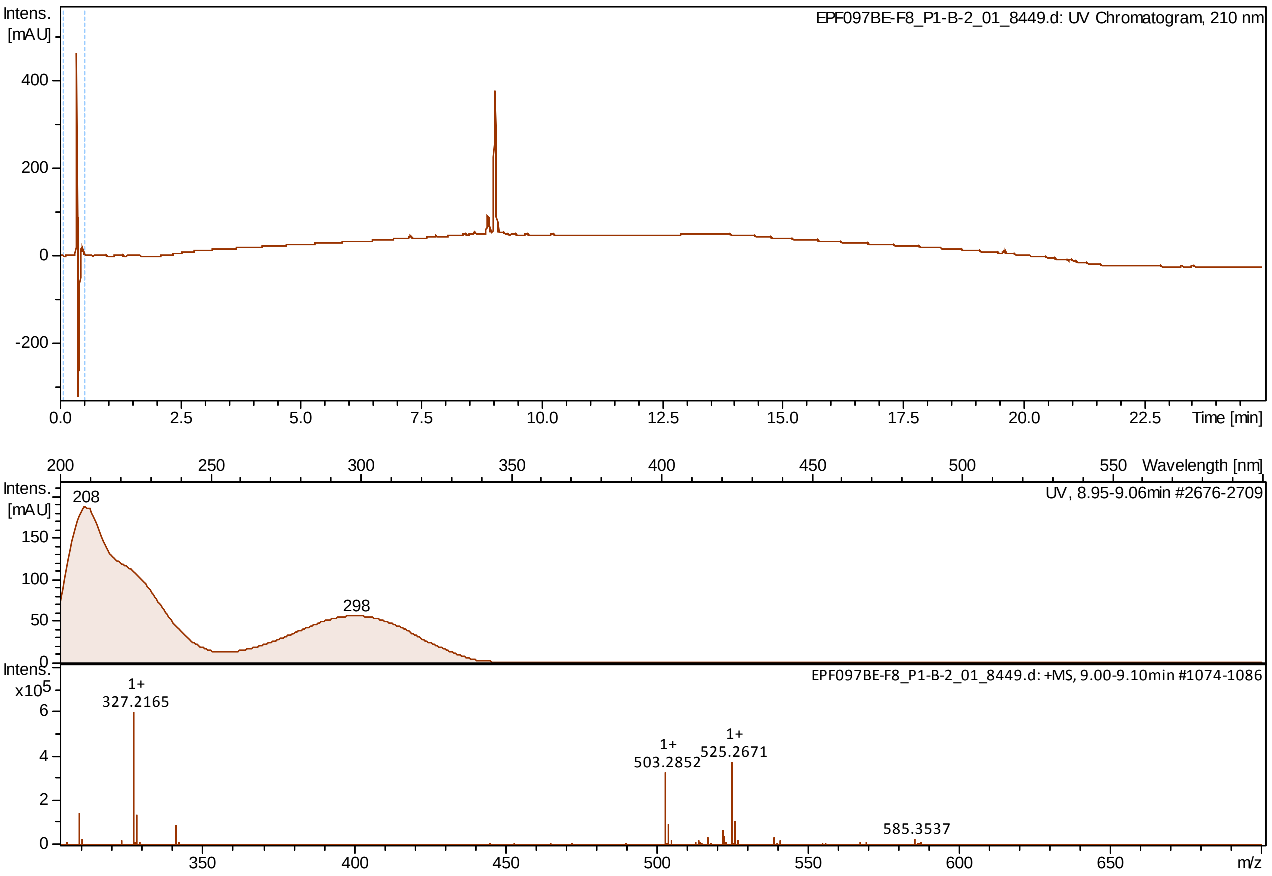

Figure S4. HR-MS chromatograms for akanthopyrone C (**3**)

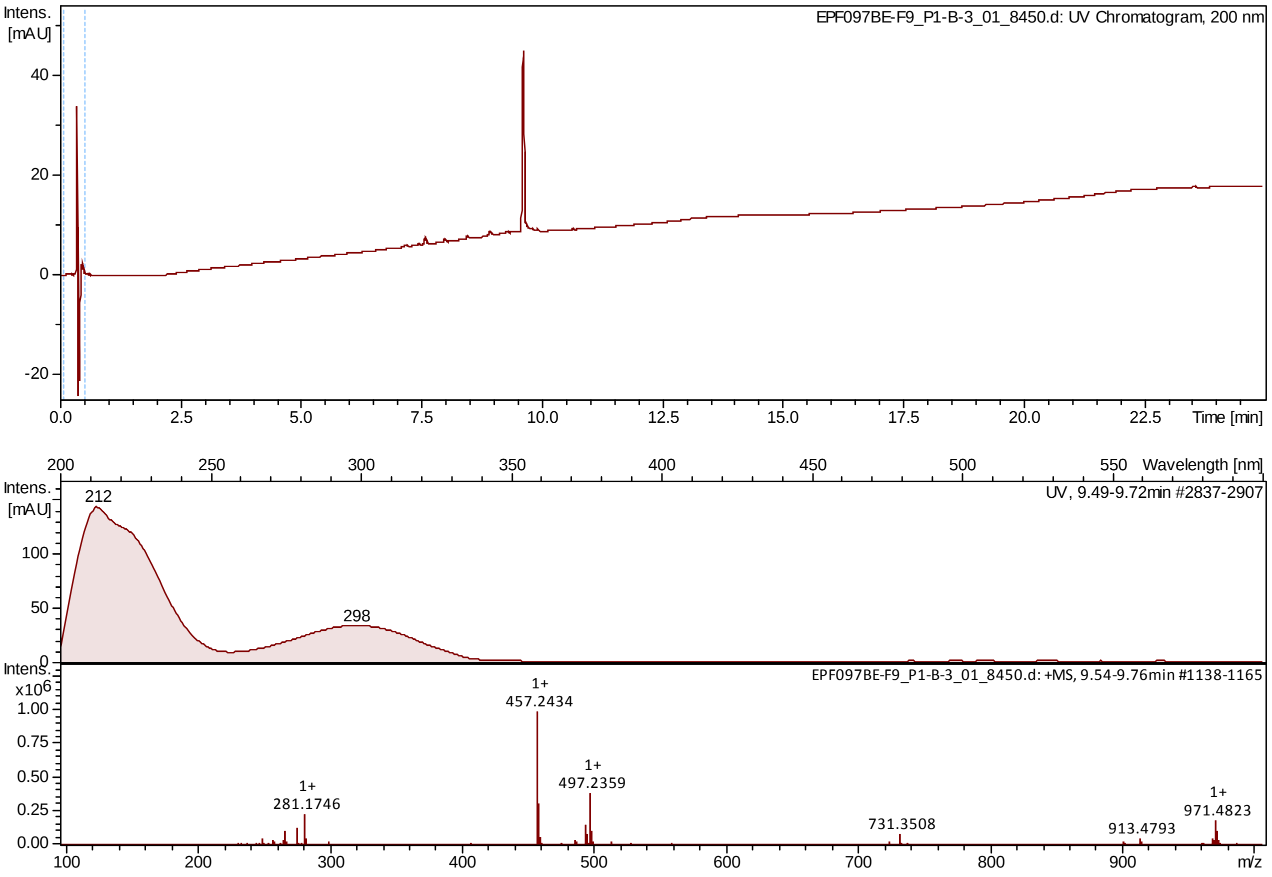

Figure S5. HR-MS chromatograms for akanthopyrone D (**4**)

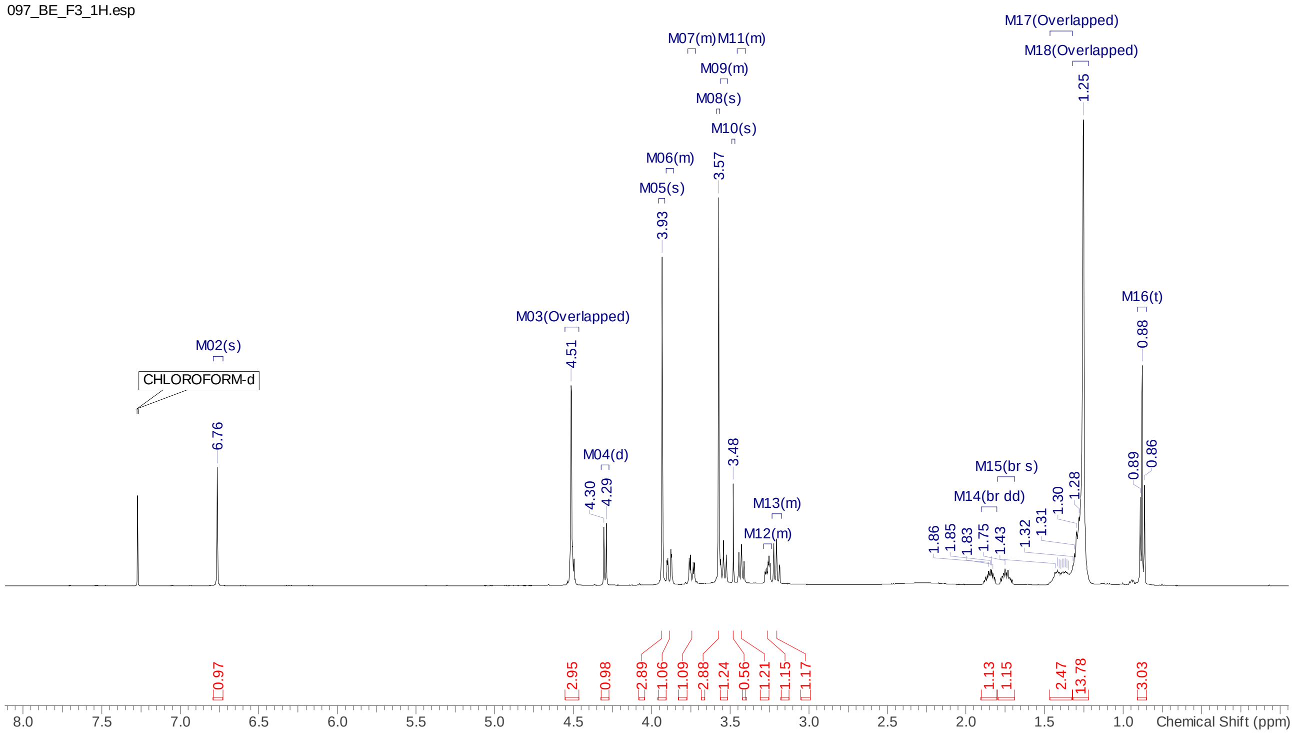

Figure S6. ^{1}H NMR spectrum for akanthopyrone A (**1**) (500 MHz, $CDCl_3$)

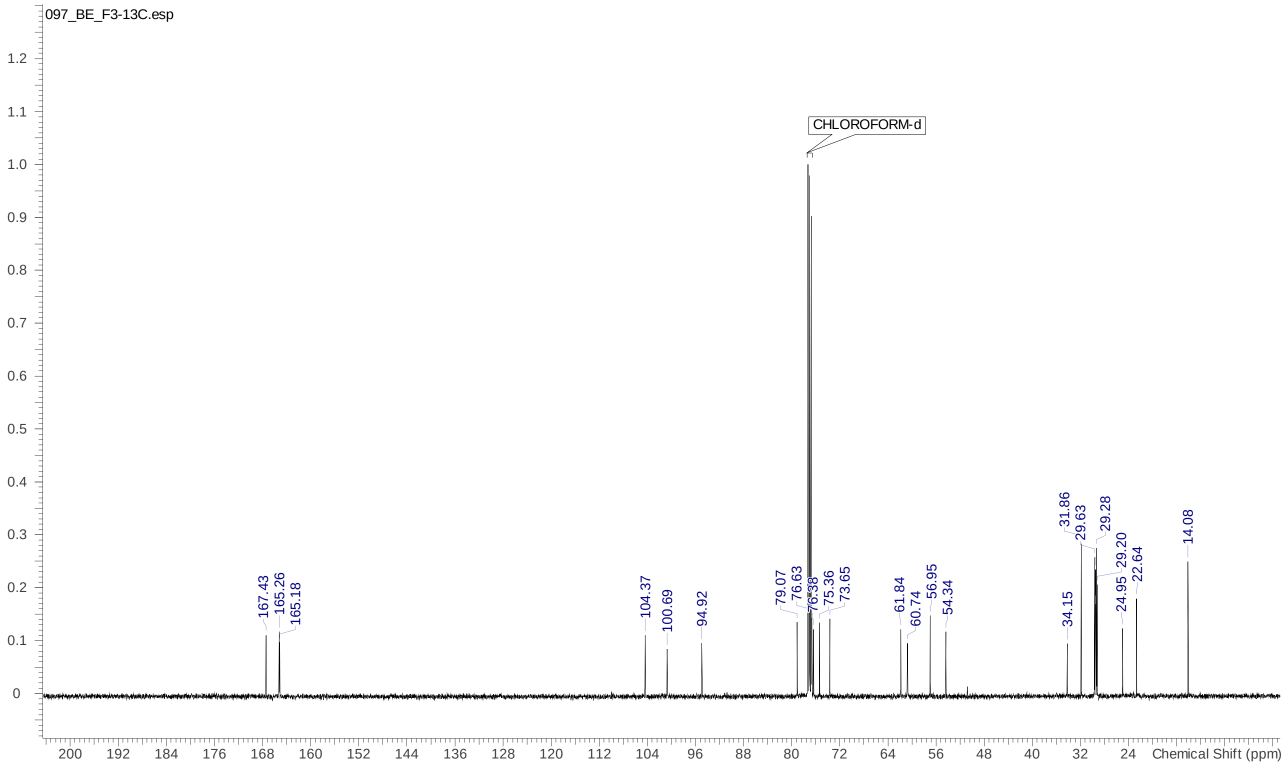

Figure S7. ^{13}C NMR spectrum for akanthopyrone A (**1**) (125 MHz, $CDCl_3$)

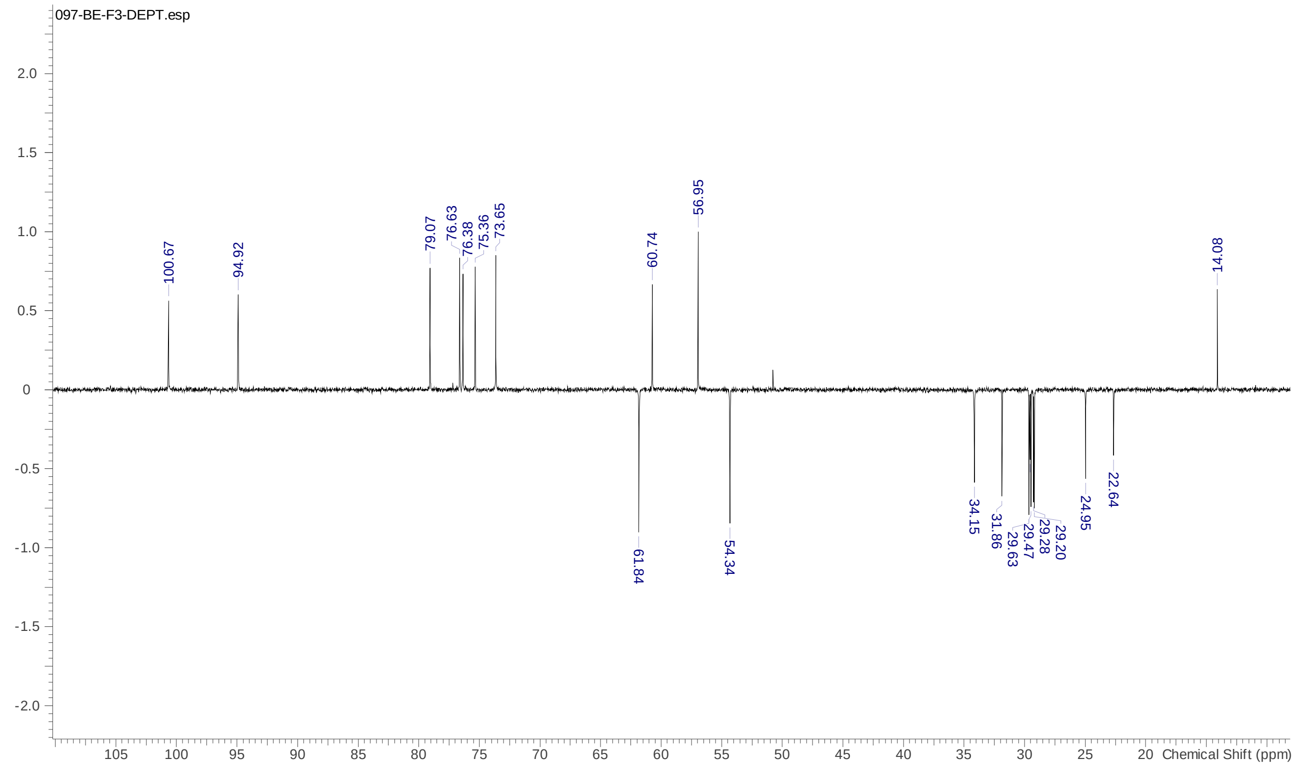

Figure S8.^{13}C DEPT-NMR spectrum for akanthopyrone A (**1**) (125 MHz, $CDCl_3$)

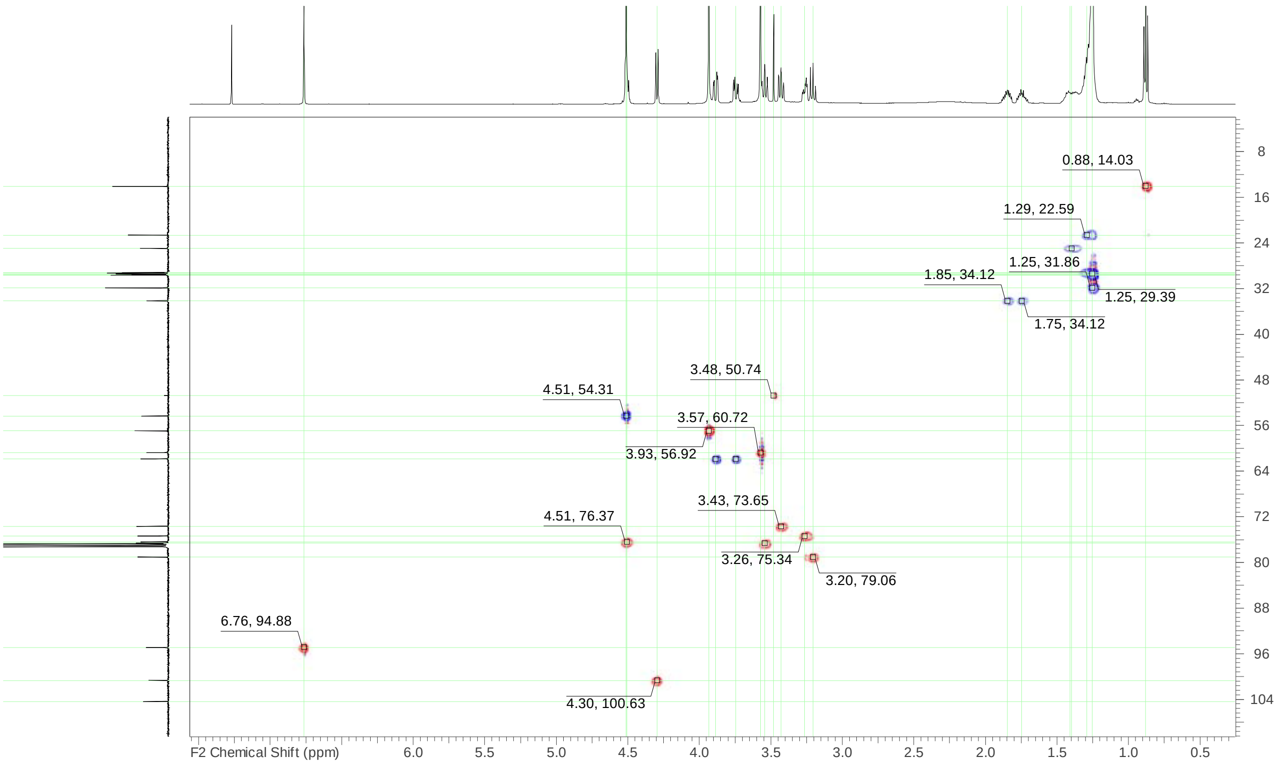

Figure S9. HSQC NMR spectrum for akanthopyrone A (**1**) (500 MHz, $CDCl_3$)

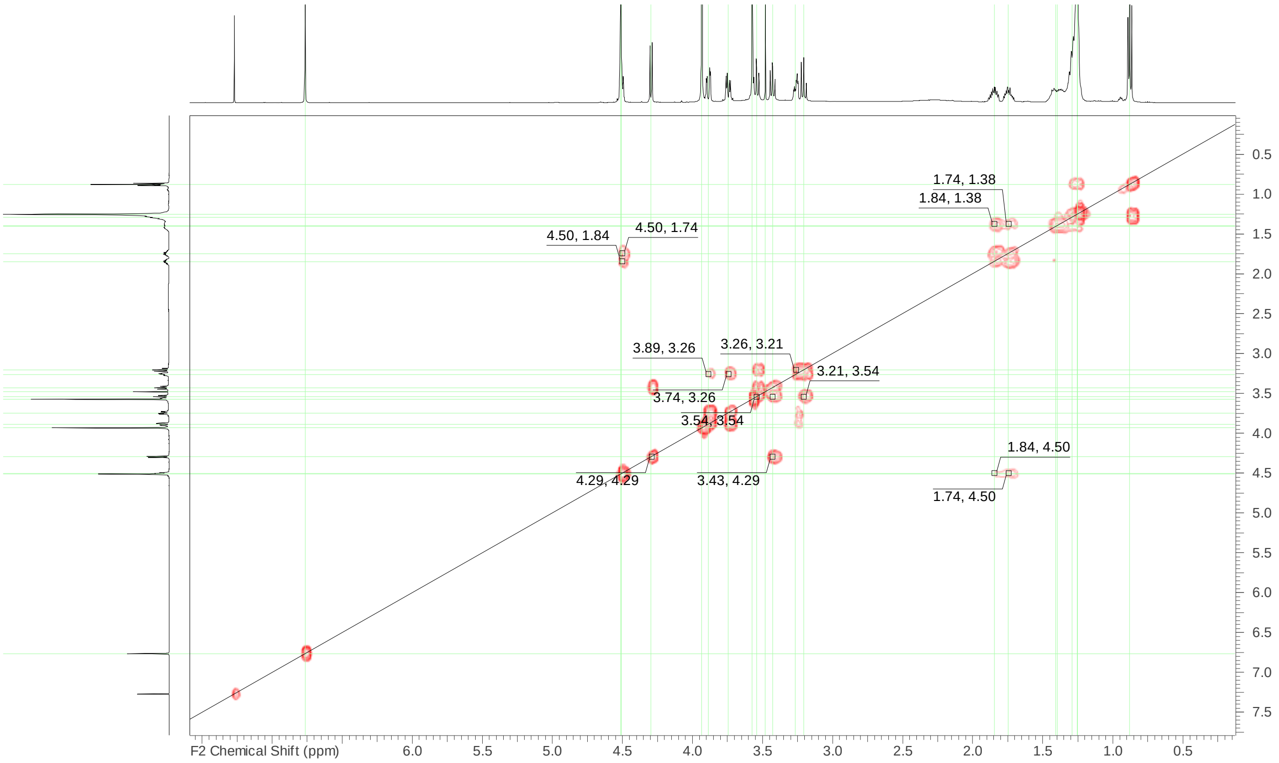

Figure S10. COSY NMR spectrum for akanthopyrone A (**1**) (500 MHz, $CDCl_3$)

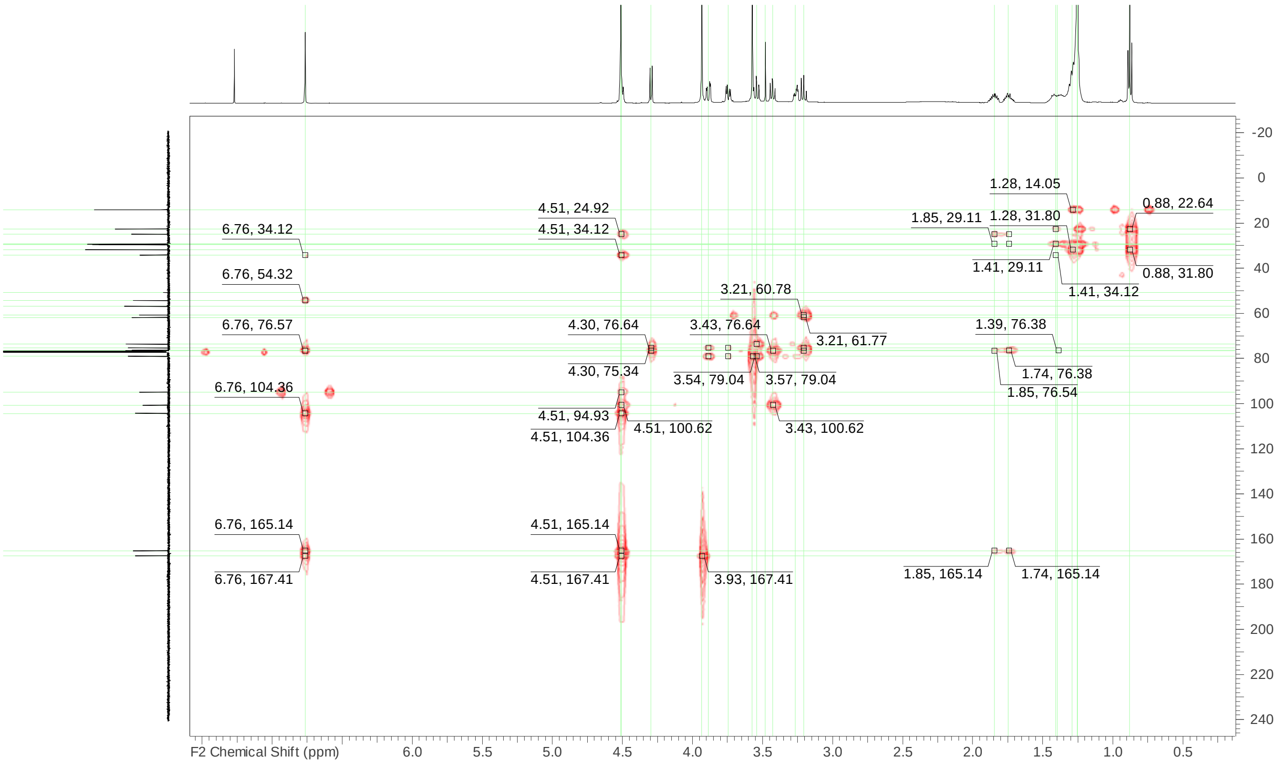

Figure S11. HMBC NMR spectrum for akanthopyrone A (**1**) (500 MHz, $CDCl_3$)

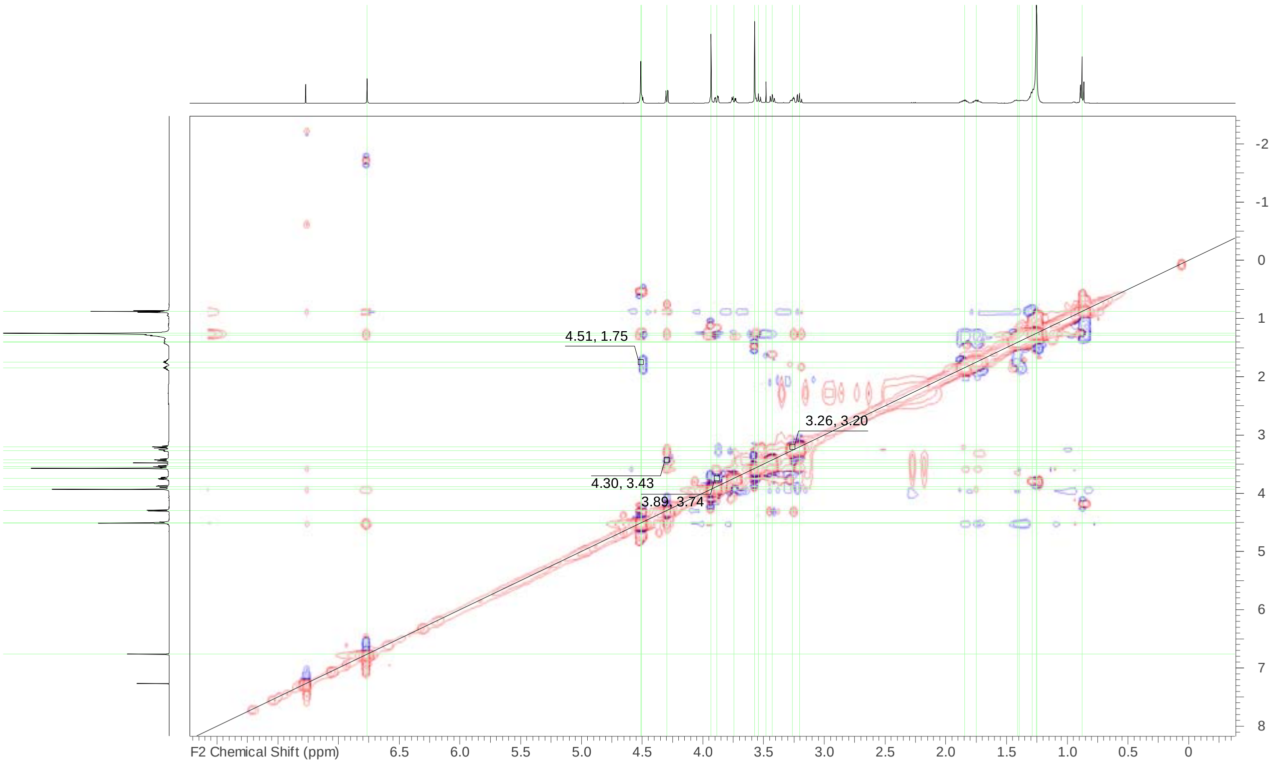

Figure S12. NOESY NMR spectrum for akanthopyrone A (**1**) (500 MHz, $CDCl_3$)

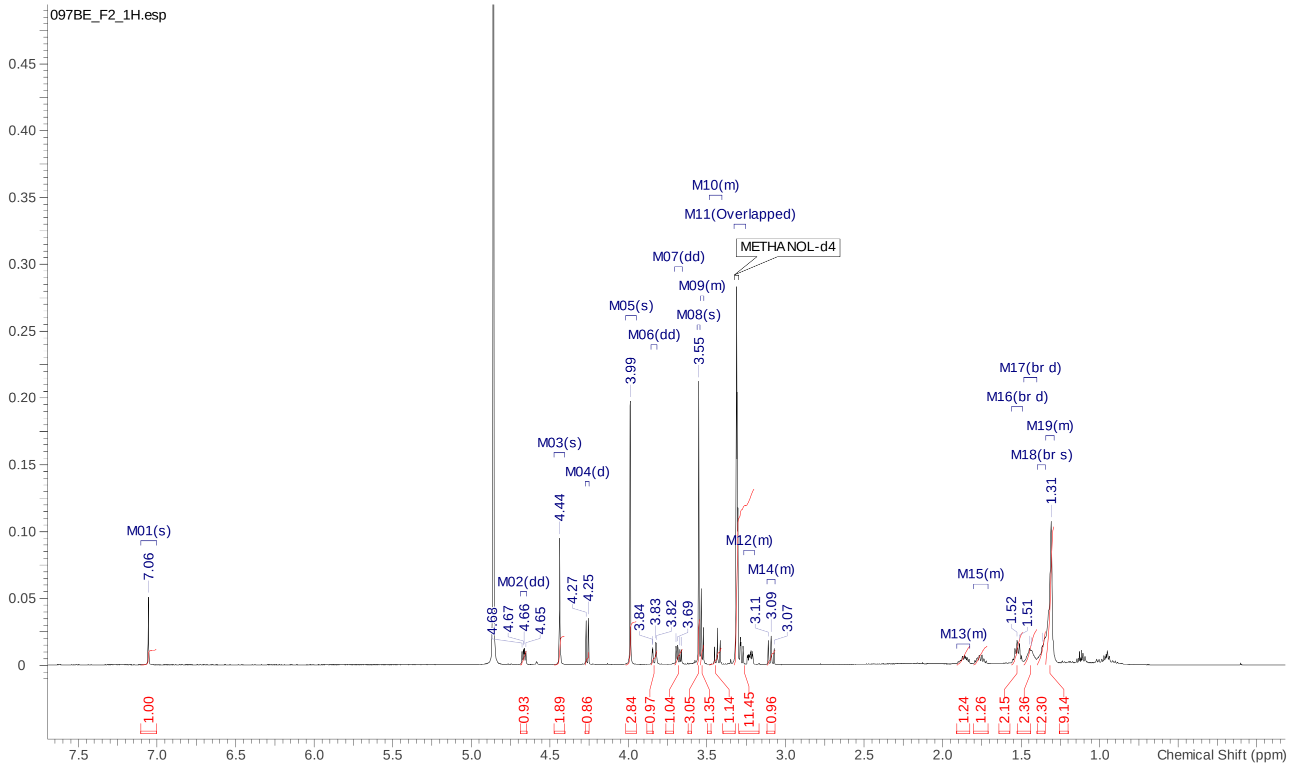

Figure S13.^{1}H NMR spectrum for akanthopyrone B (**2**) (500 MHz, CD_3OD)

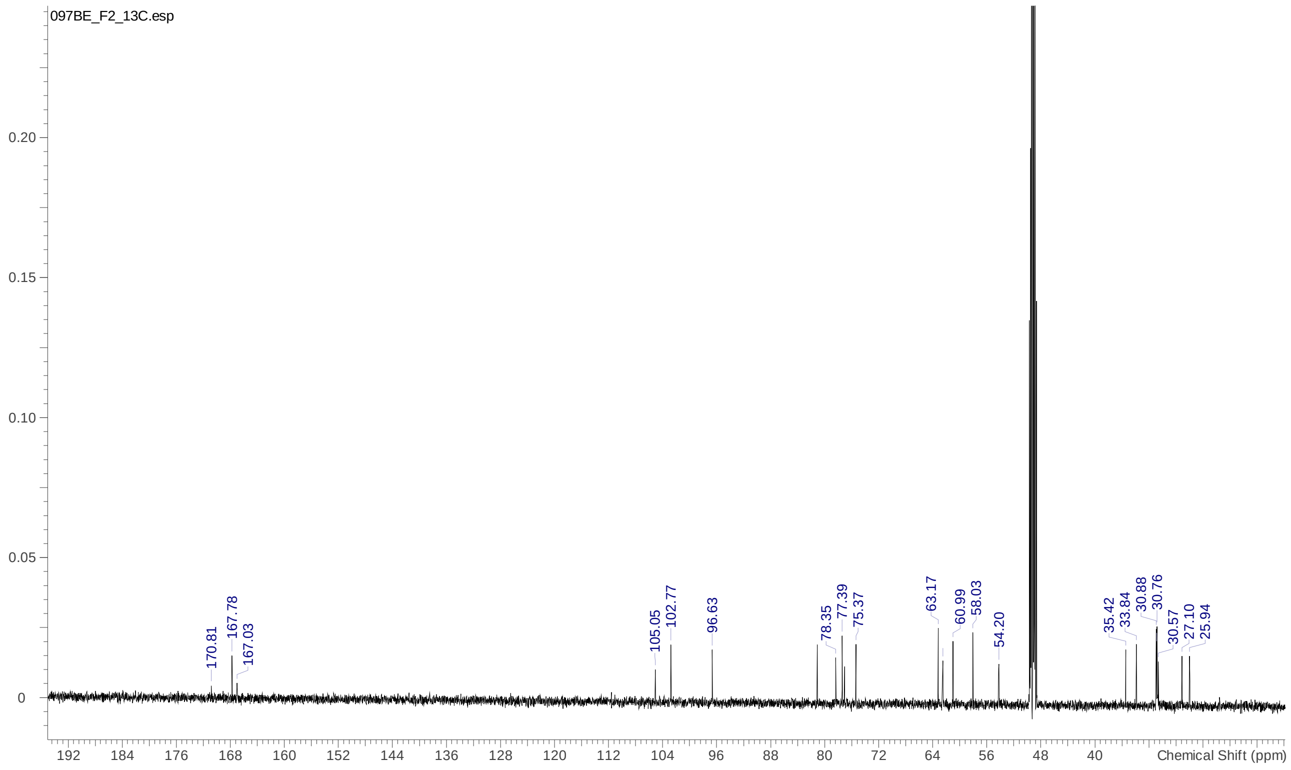

Figure S14.^{13}C NMR spectrum for akanthopyrone B (**2**) (125 MHz, CD_3OD)

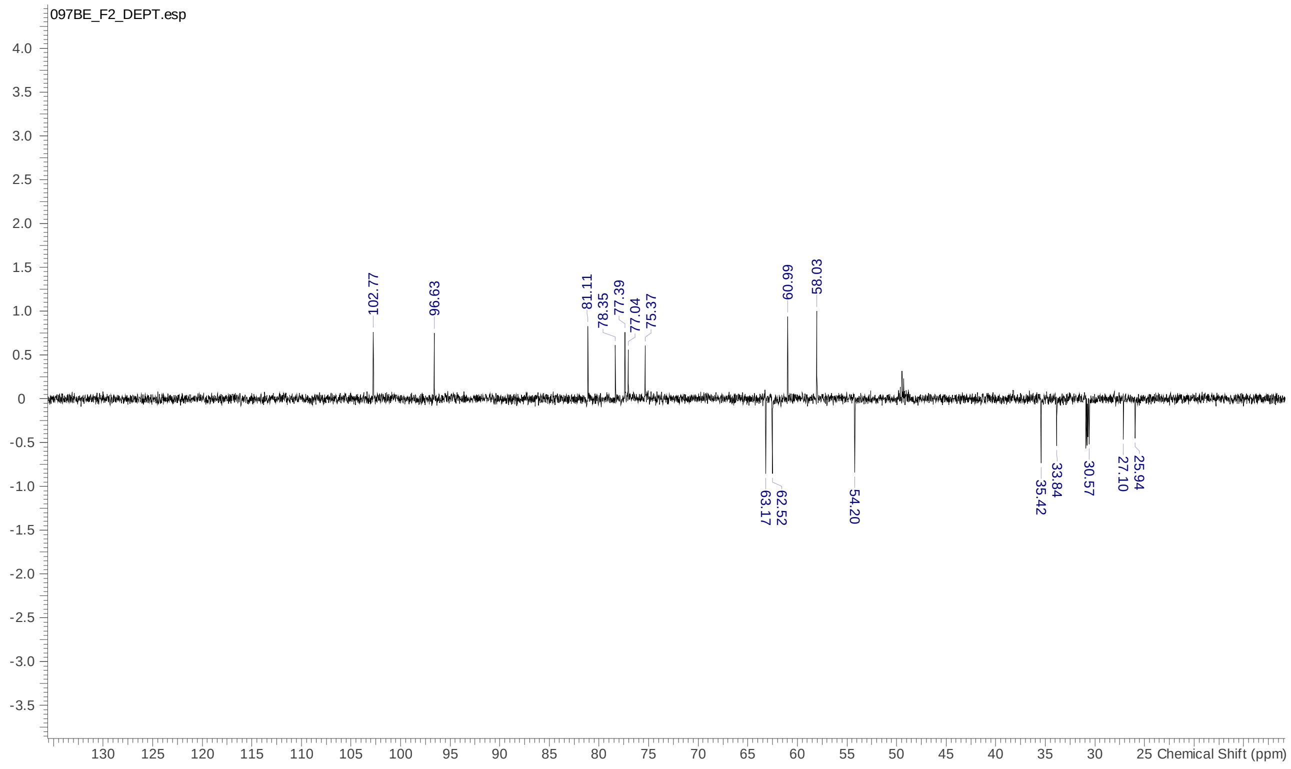

Figure S15.^{13}C DEPT-NMR spectrum for akanthopyrone B (**2**) (125 MHz, CD_3OD)

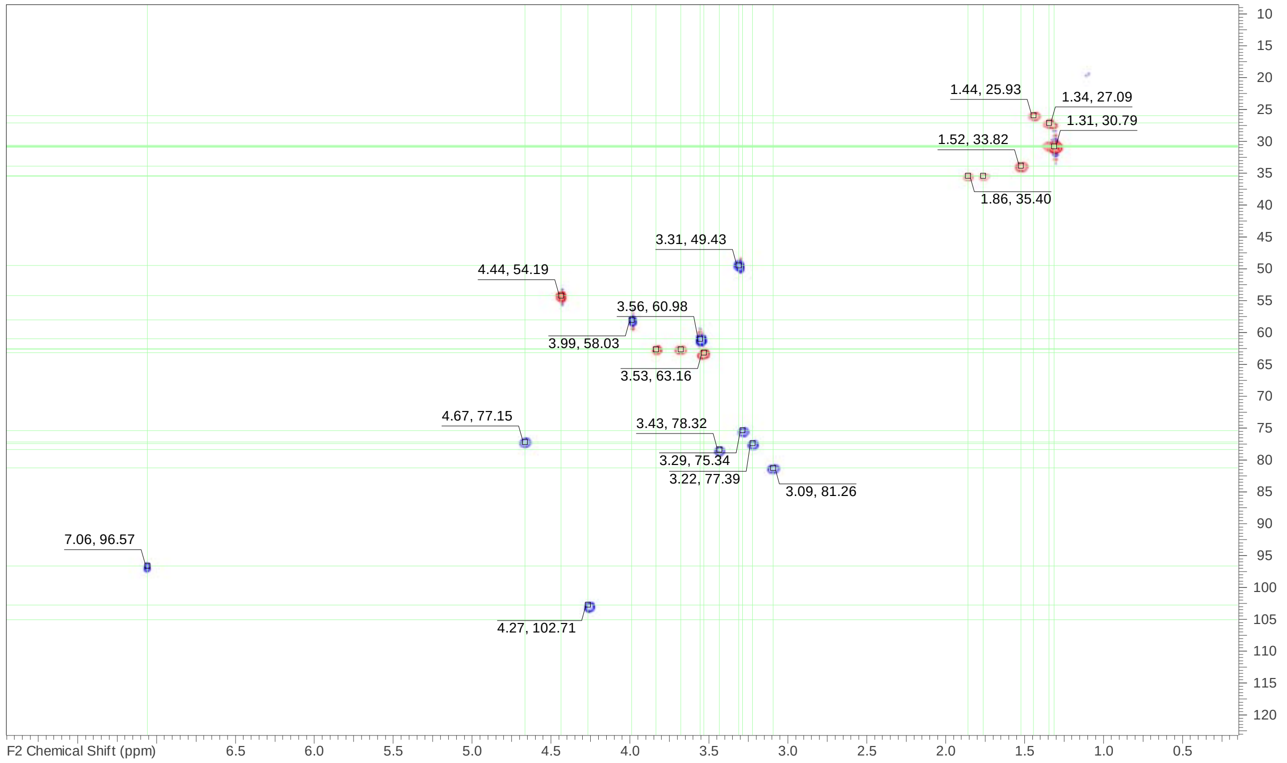

Figure S16. HSQC NMR spectrum for akanthopyrone B (**2**) (500 MHz, CD_3OD)

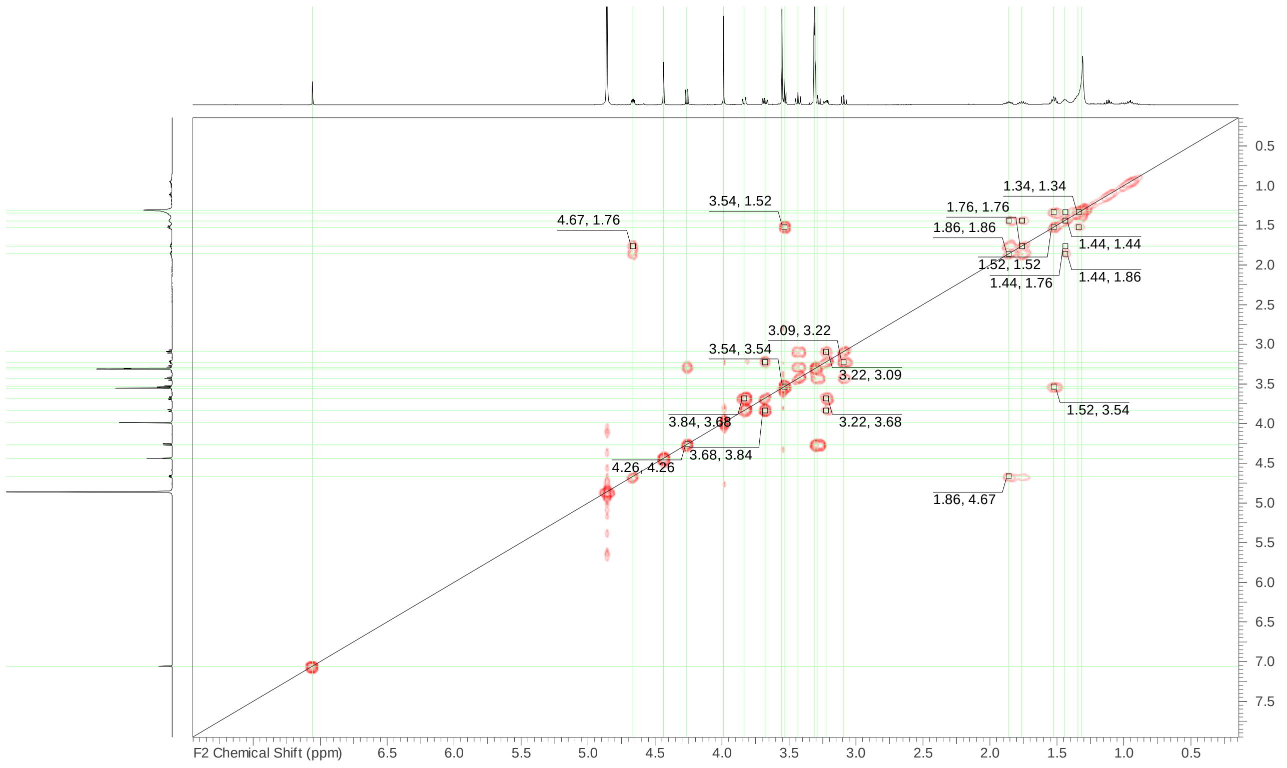

Figure S17. COSY NMR spectrum for akanthopyrone B (**2**) (500 MHz, CD_3OD)

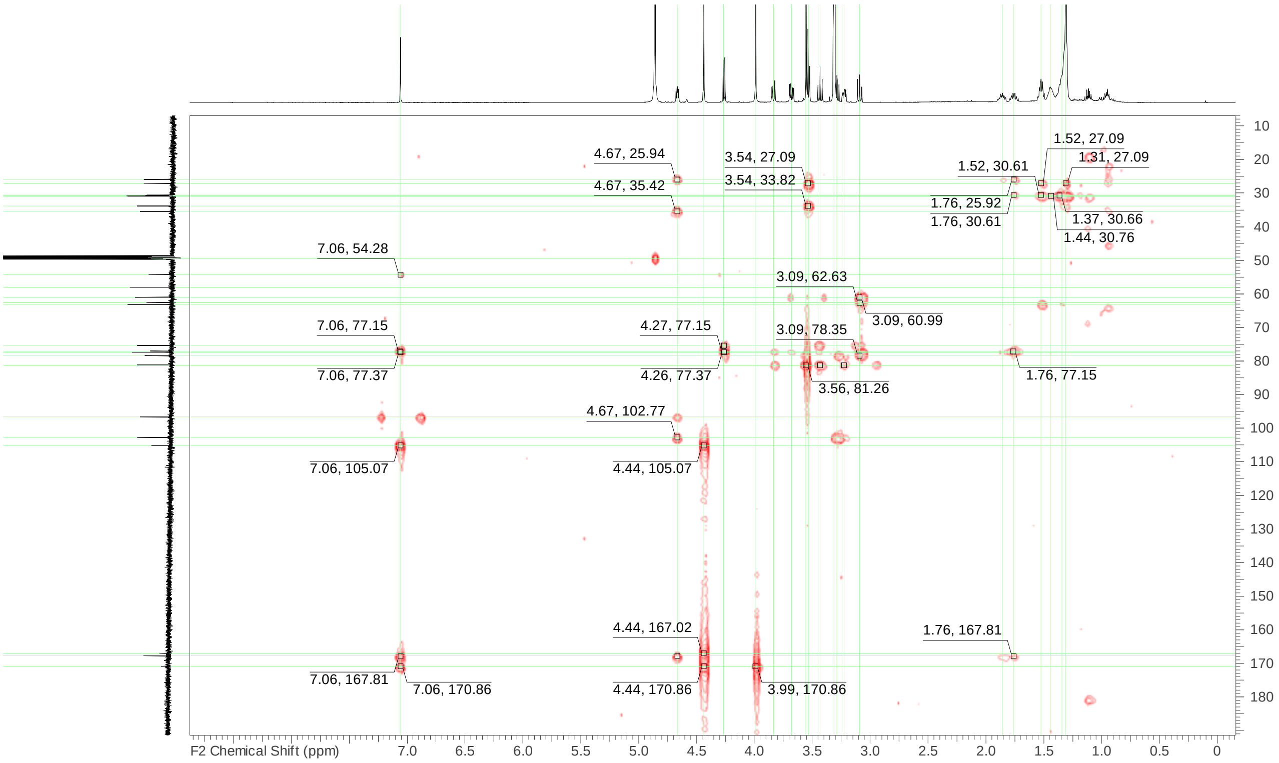

Figure S18. HMBC NMR spectrum for akanthopyrone B (**2**) (500 MHz, CD_3OD)

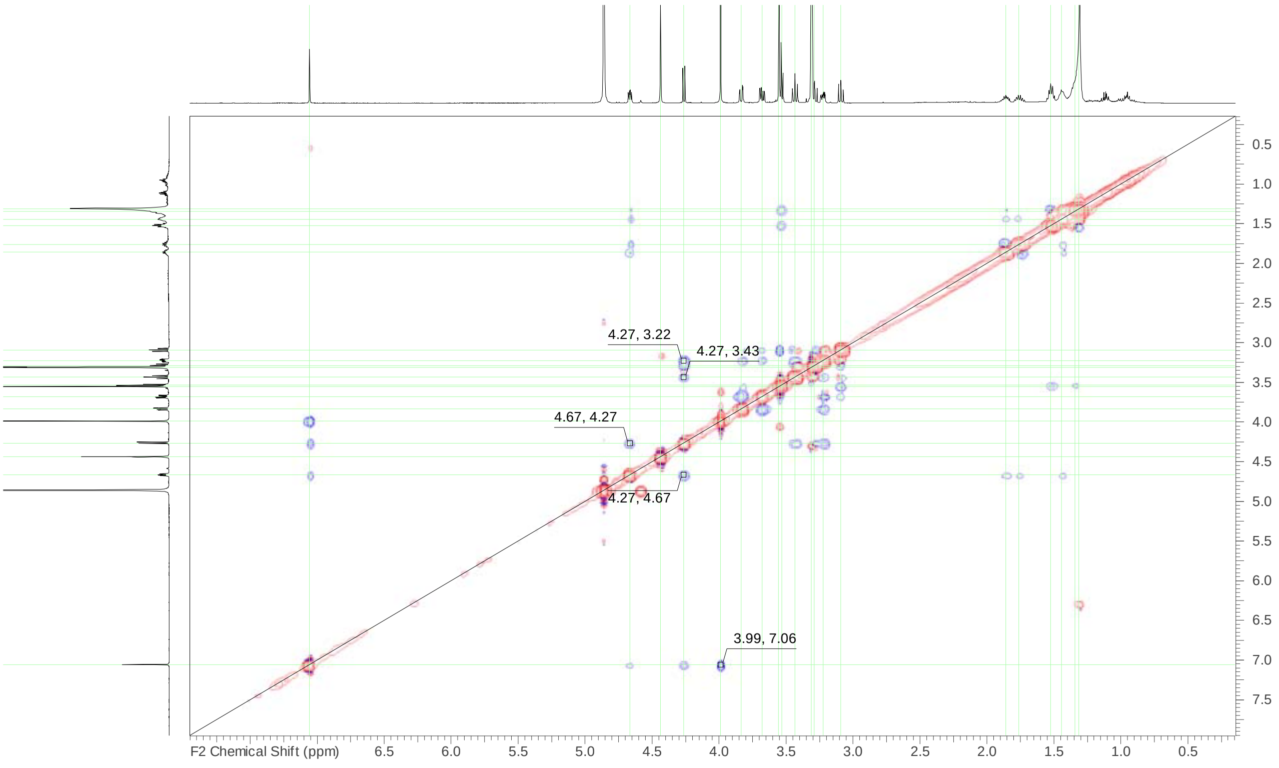

Figure S19. NOESY NMR spectrum for akanthopyrone B (**2**) (500 MHz, CD_3OD)

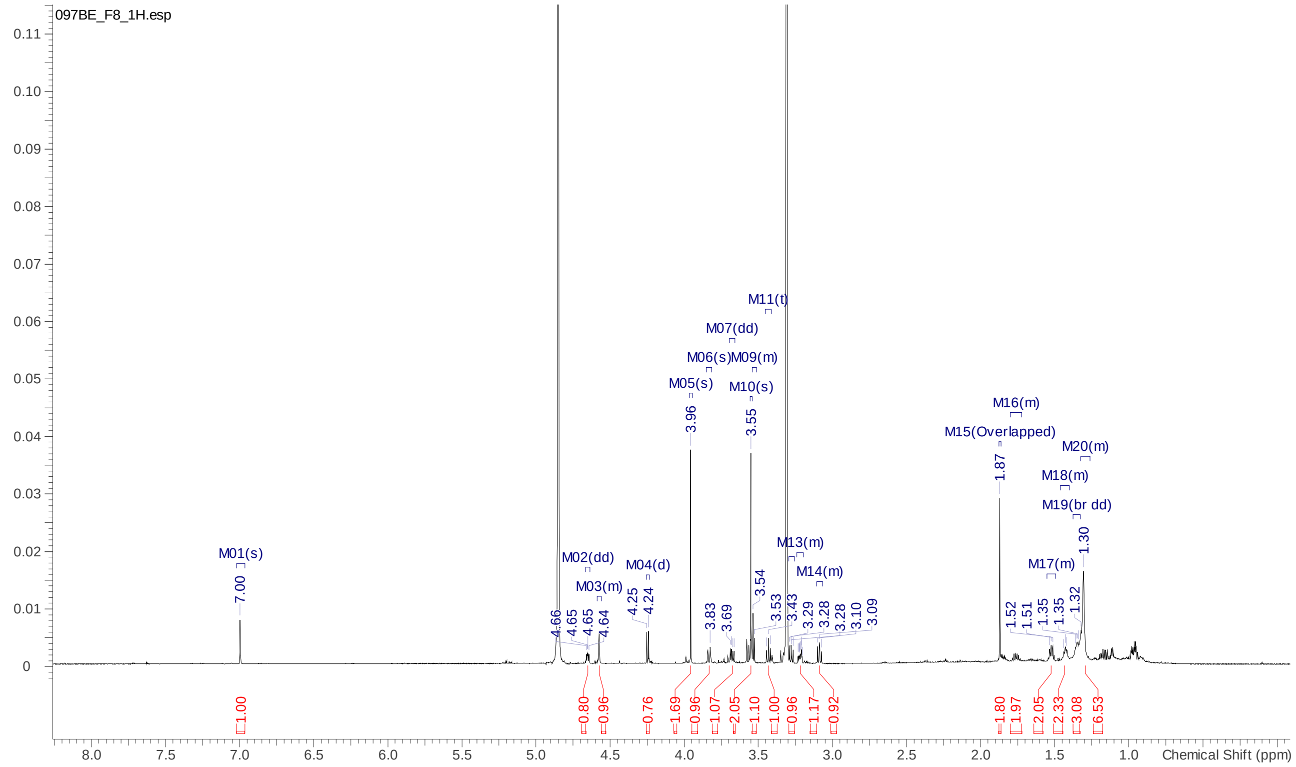

Figure S20. ^{1}H NMR spectrum for akanthopyrone C (**3**) (700 MHz, CD_3OD)

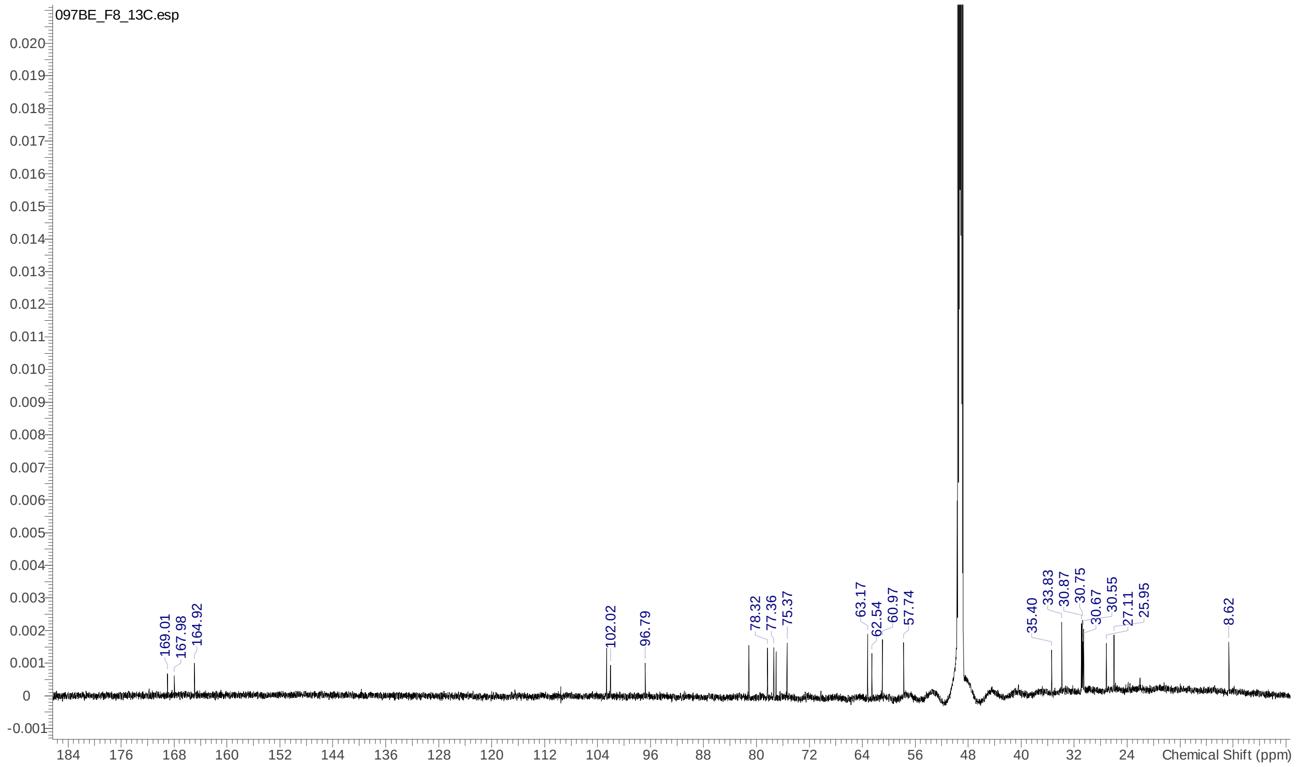

Figure S21.^{13}C NMR spectrum for akanthopyrone C (**3**) (175 MHz, CD_3OD)

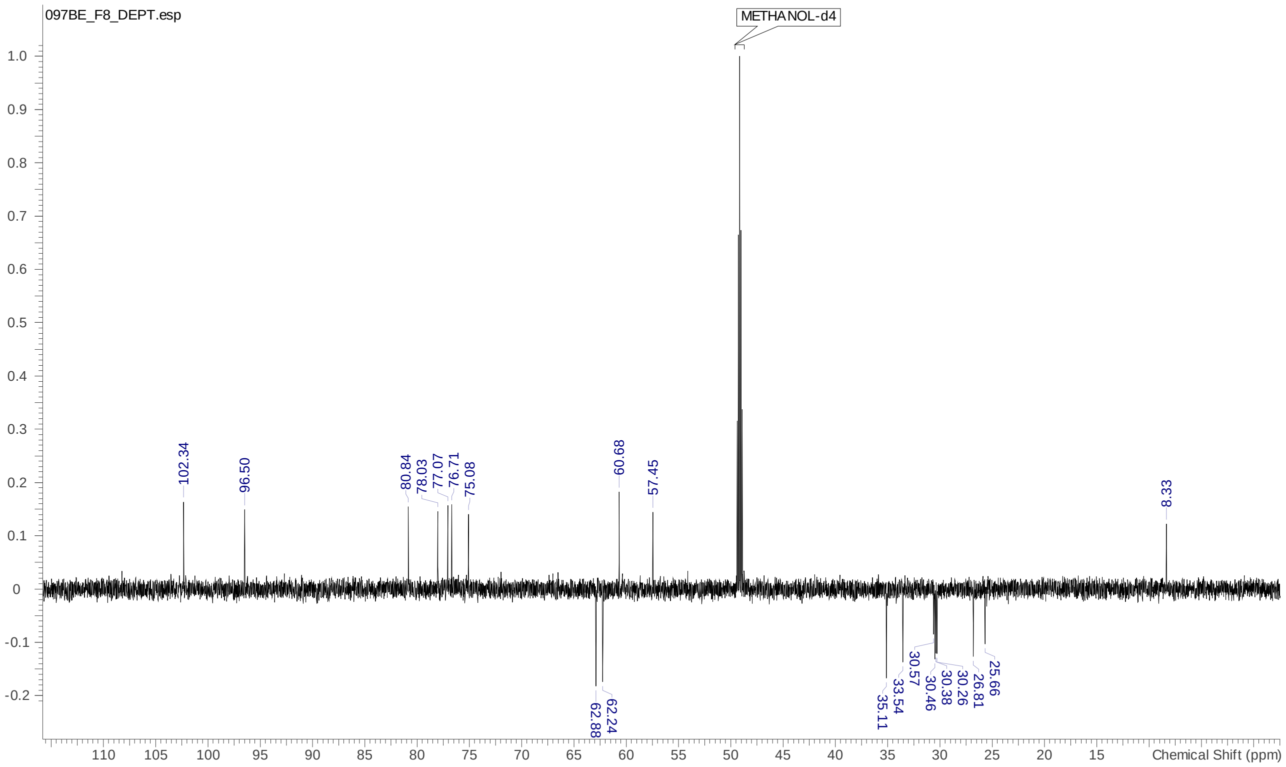

Figure S22.^{13}C DEPT-NMR spectrum for akanthopyrone C (**3**) (175 MHz, CD_3OD)

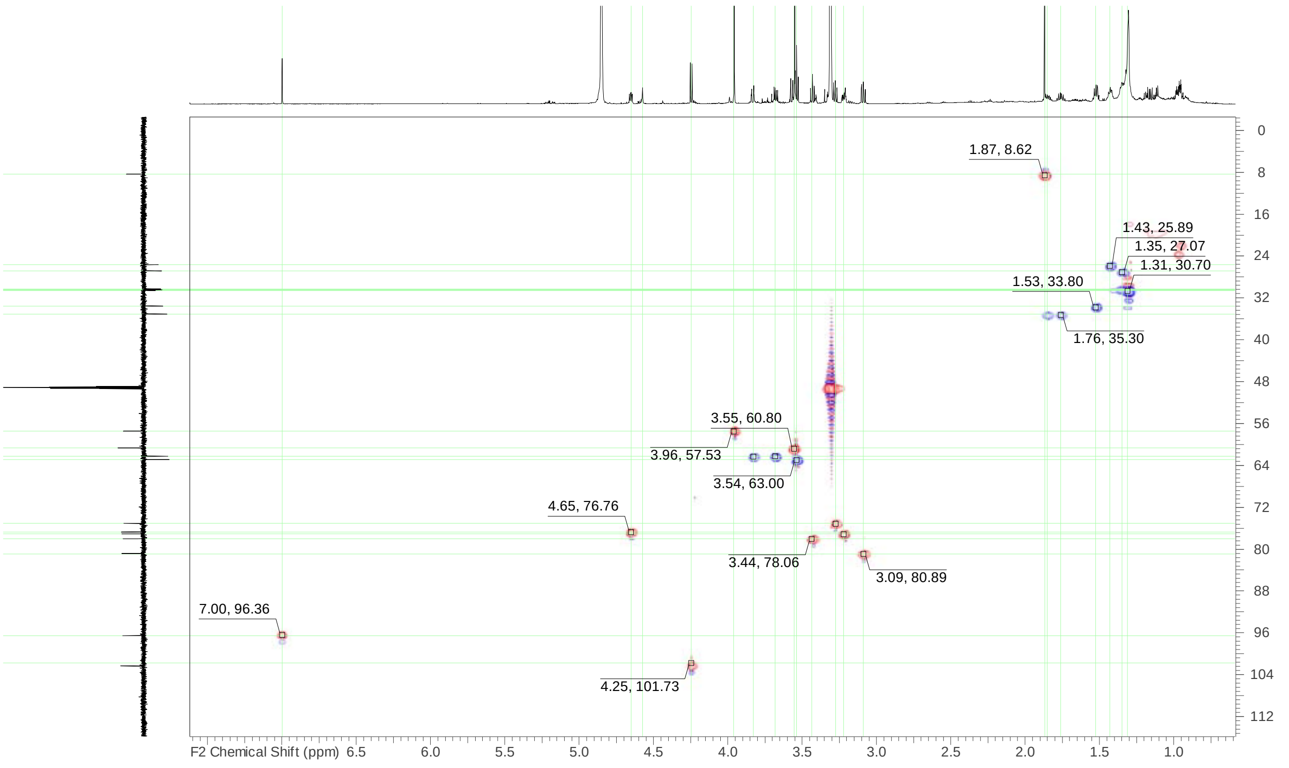

Figure S23. HSQC NMR spectrum for akanthopyrone C (**3**) (700 MHz, CD_3OD)

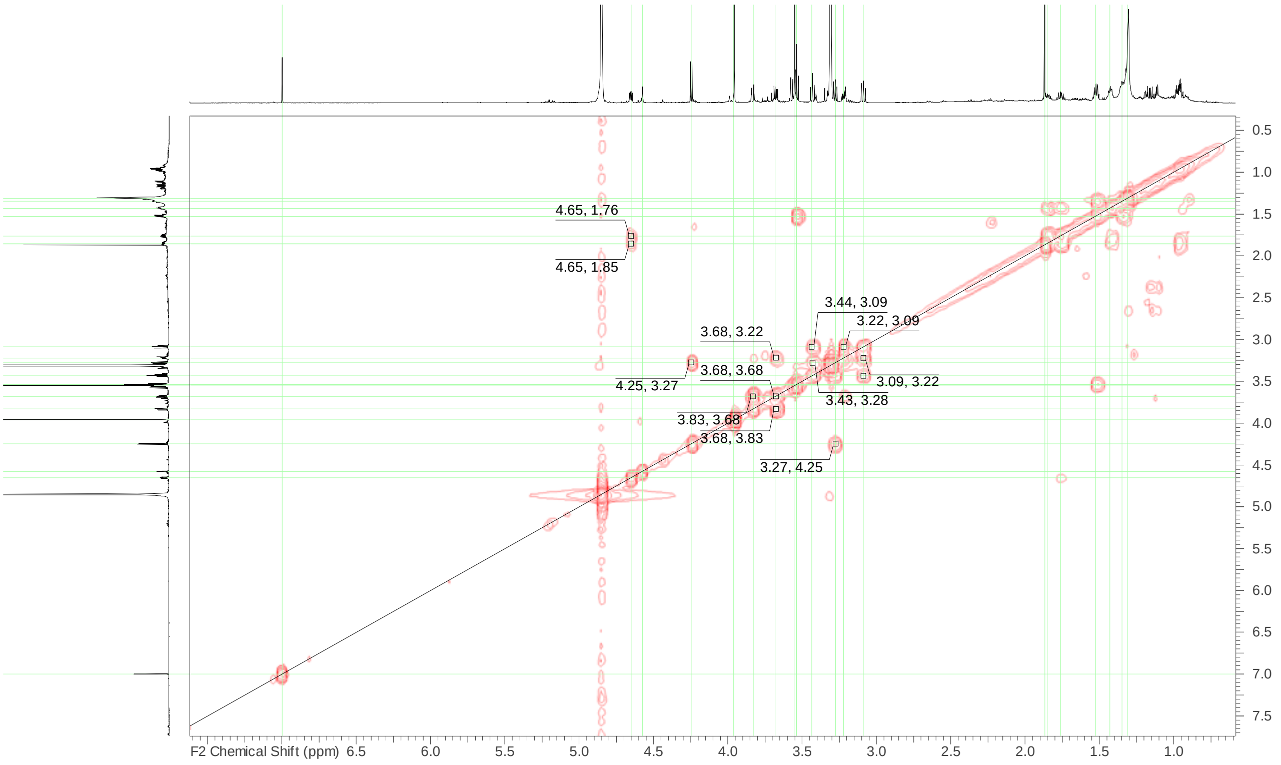

Figure S24. COSY NMR spectrum for akanthopyrone C (**3**) (700 MHz, CD_3OD)

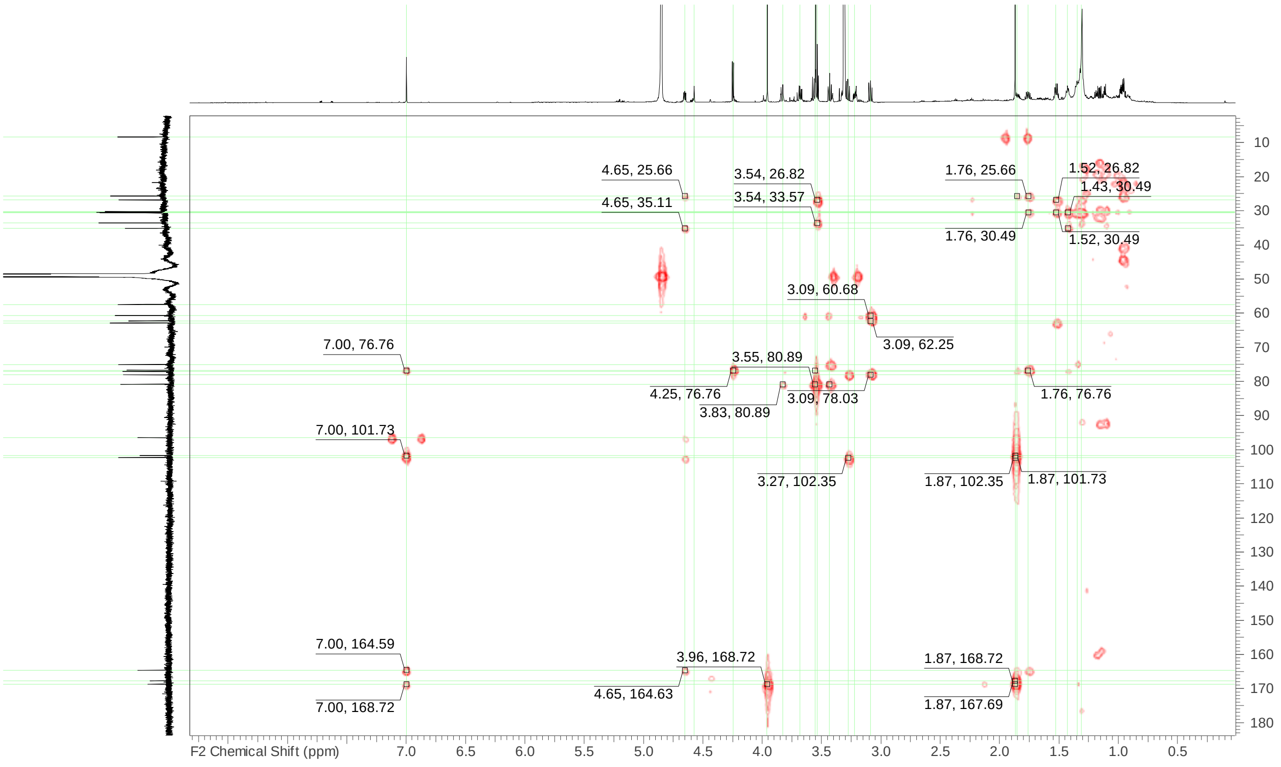

Figure S25. HMBC NMR spectrum for akanthopyrone C (**3**) (700 MHz, CD_3OD)

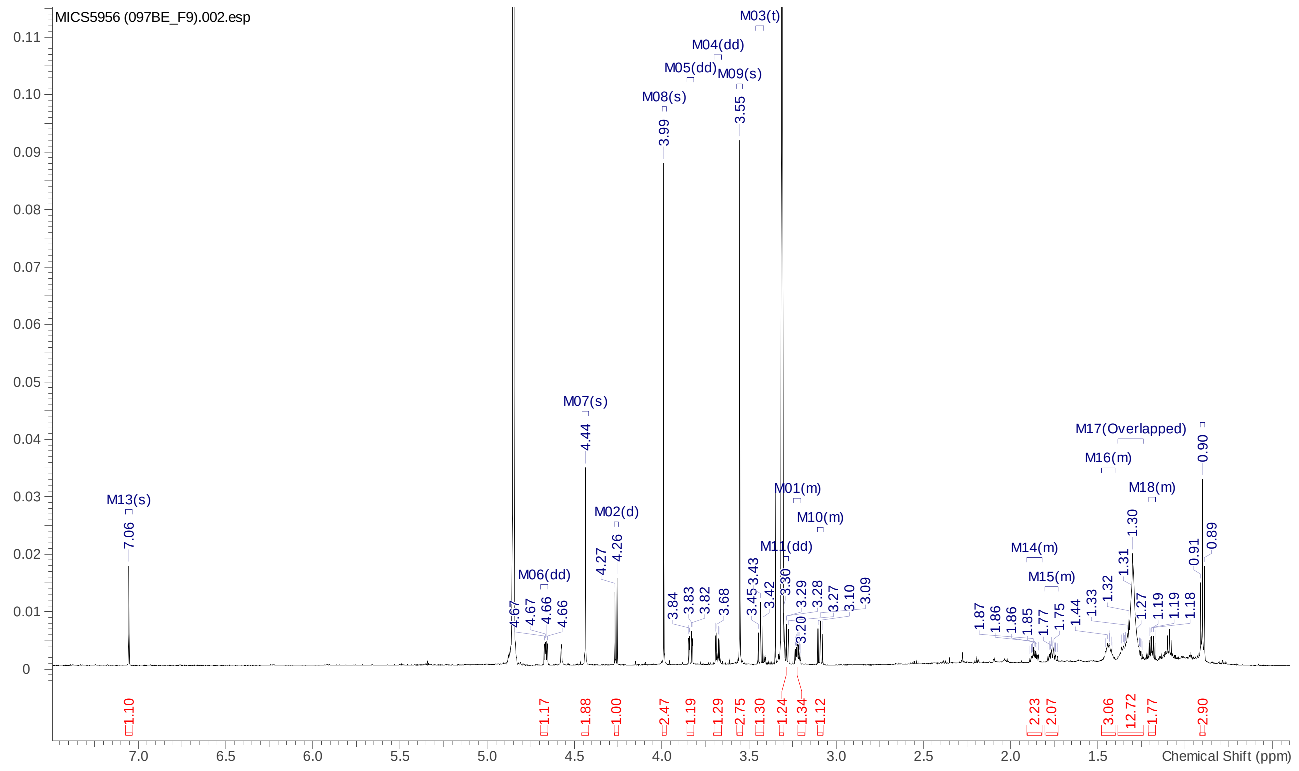

Figure S26.^{1}H NMR spectrum for akanthopyrone D (**4**) (700 MHz, CD_3OD)

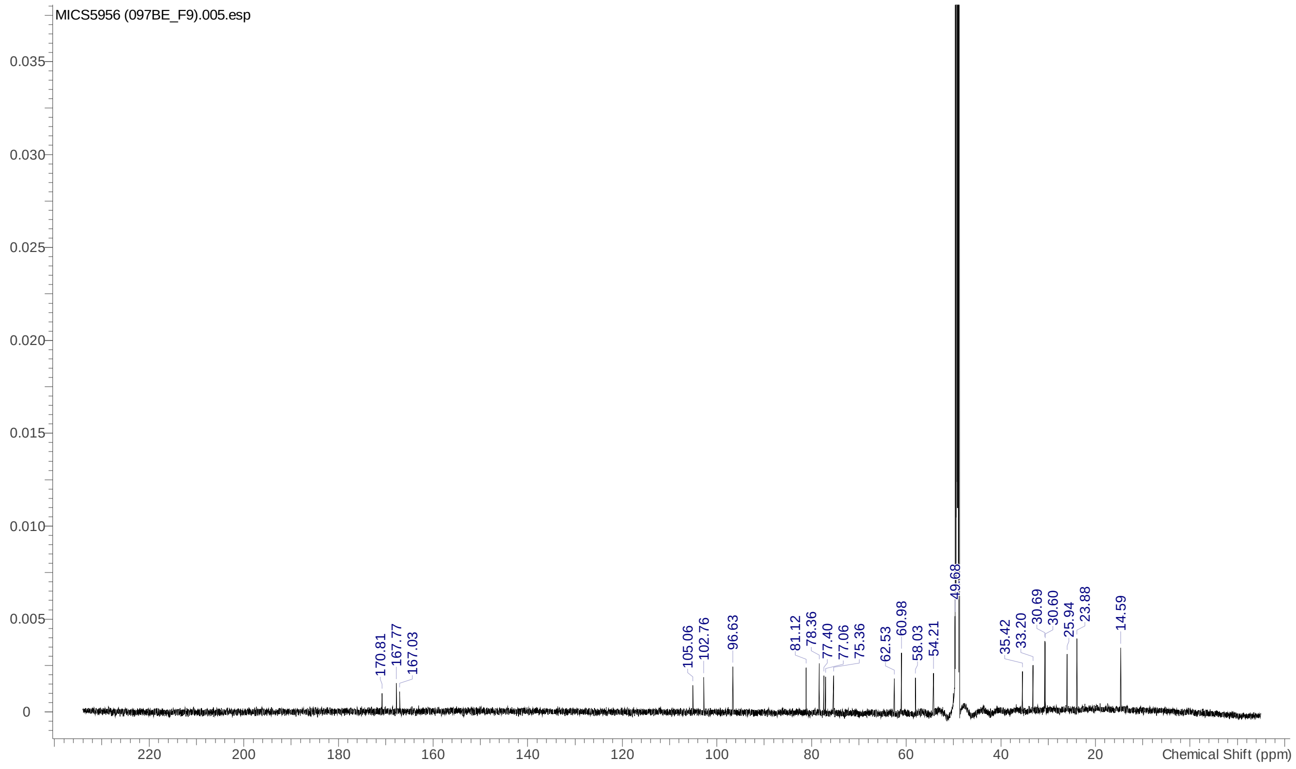

Figure S27. ^{13}C NMR spectrum for akanthopyrone D (**4**) (175 MHz, CD_3OD)

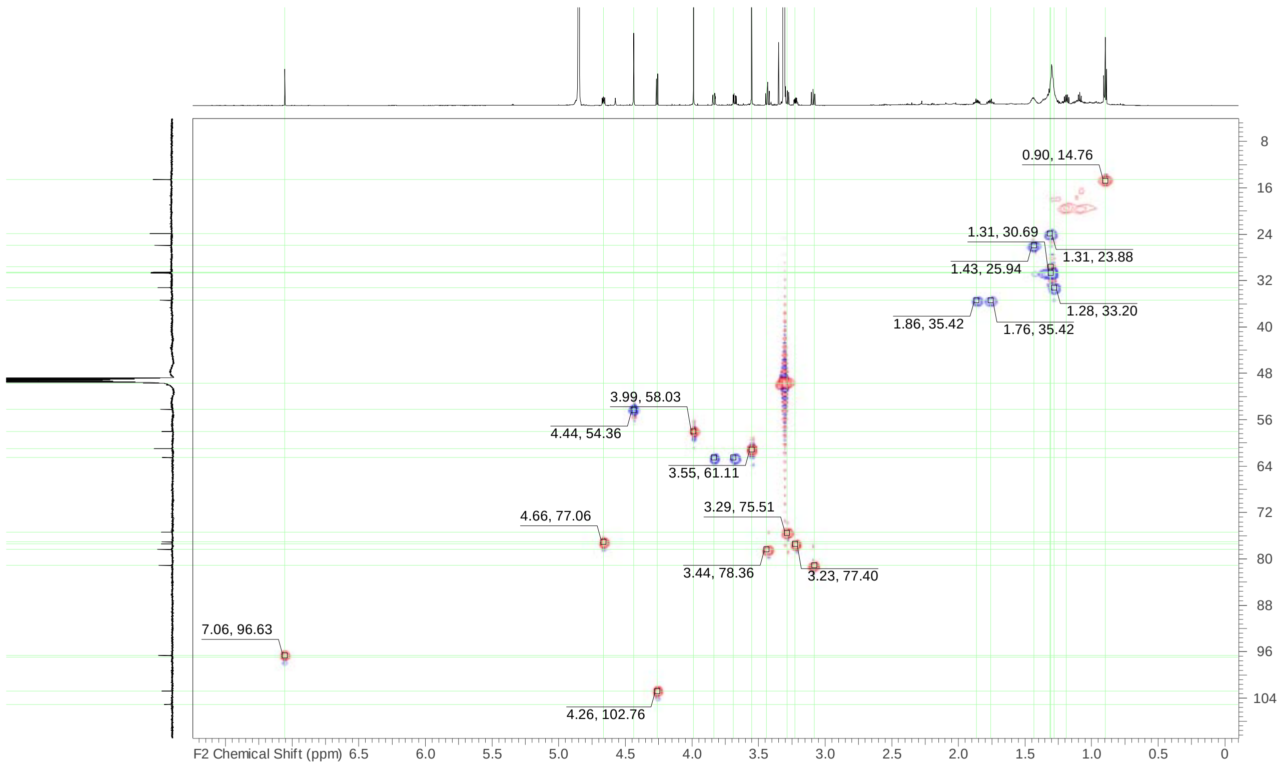

Figure S28. HSQC NMR spectrum for akanthopyrone D (**4**) (700 MHz, CD_3OD)

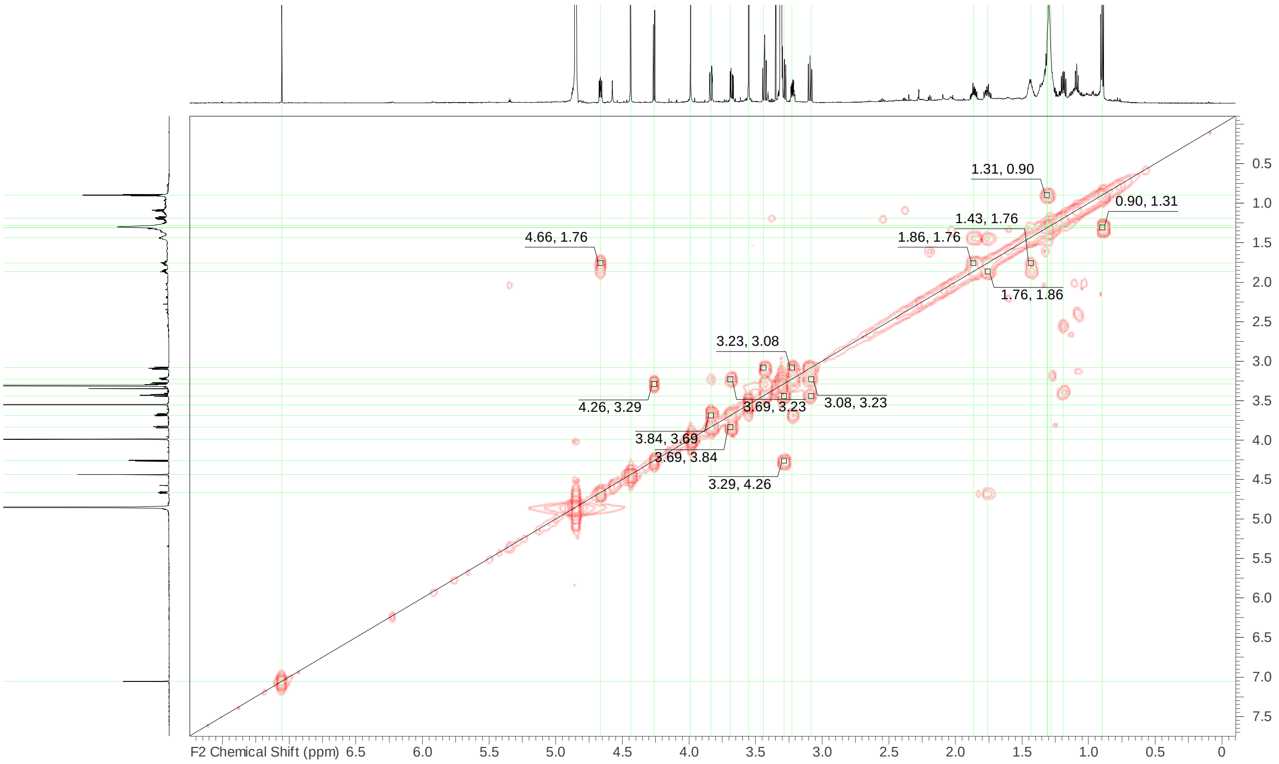

Figure S29. COSY NMR spectrum for akanthopyrone D (**4**) (700 MHz, CD_3OD)

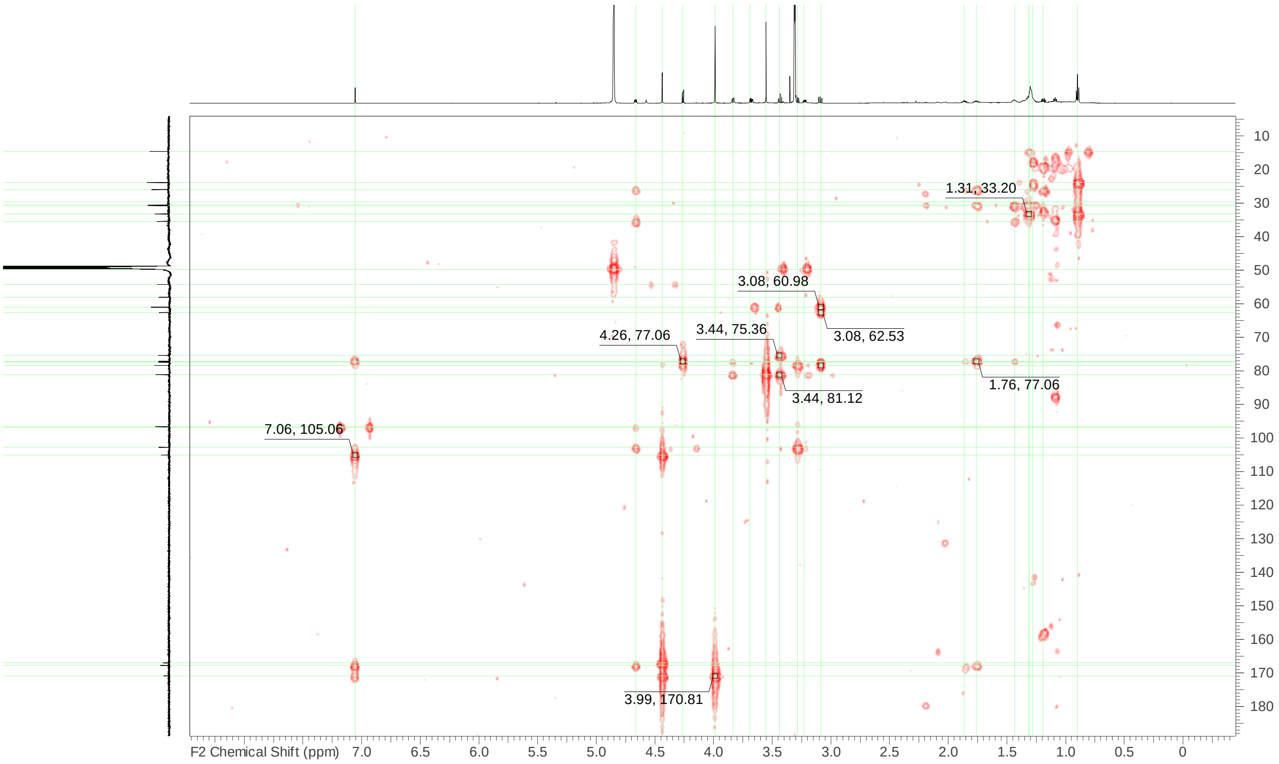

Figure S30. HMBC NMR spectrum for akanthopyrone D (**4**) (700 MHz, CD_3OD)

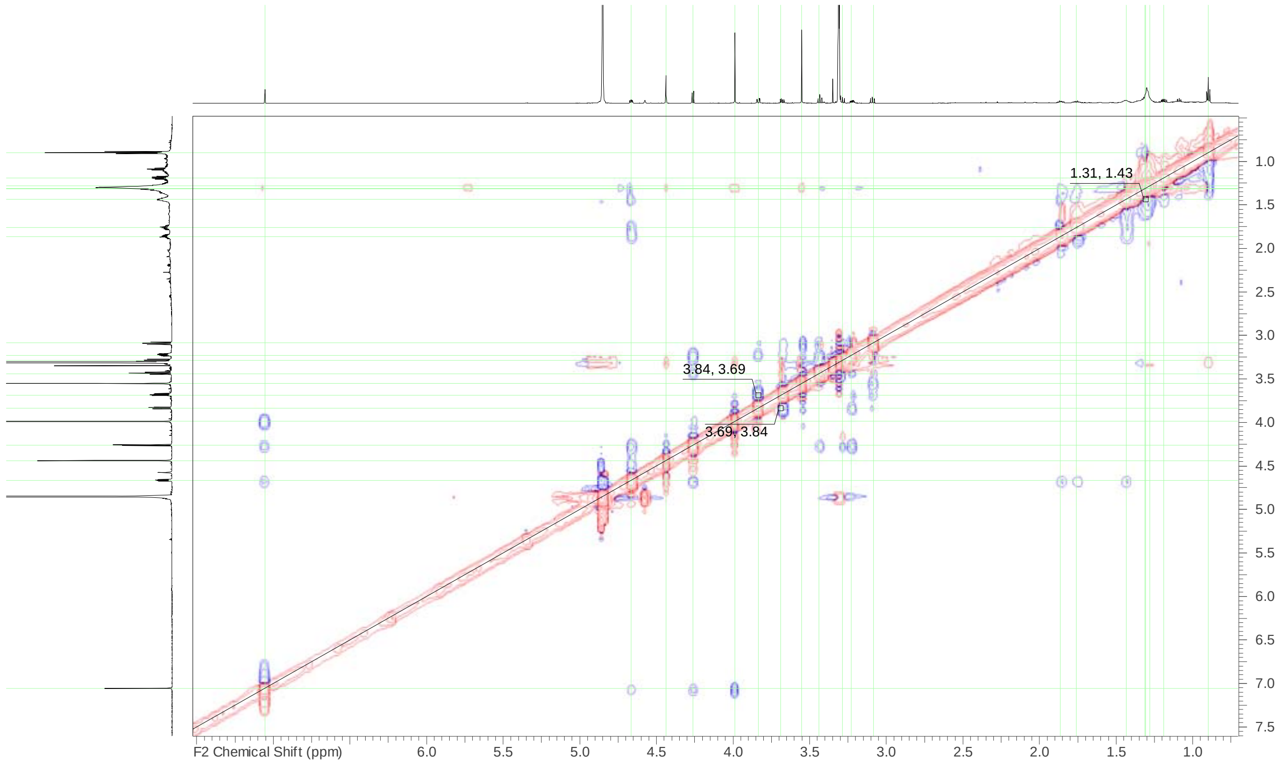

Figure S31. NOESY NMR spectrum for akanthopyrone D (**4**) (700 MHz, CD_3OD)
